# Extensions of MADM (Mosaic Analysis with Double Markers) in Mice

**DOI:** 10.1371/journal.pone.0033332

**Published:** 2012-03-27

**Authors:** Bosiljka Tasic, Kazunari Miyamichi, Simon Hippenmeyer, Vardhan S. Dani, Hong Zeng, William Joo, Hui Zong, Yanru Chen-Tsai, Liqun Luo

**Affiliations:** 1 Department of Biology, Howard Hughes Medical Insitute, Stanford University, California, United States of America; 2 Transgenic Facility, Stanford Cancer Center, Stanford University School of Medicine, California, United States of America; 3 Institute of Molecular Biology, University of Oregon, Eugene, Oregon, United States of America; 4 Neurosciences Program, Stanford University, California, United States of America; Columbia University, United States of America

## Abstract

Mosaic Analysis with Double Markers (MADM) is a method for generating genetically mosaic mice, in which sibling mutant and wild-type cells are labeled with different fluorescent markers. It is a powerful tool that enables analysis of gene function at the single cell level *in vivo*. It requires transgenic cassettes to be located between the centromere and the mutation in the gene of interest on the same chromosome. Here we compare procedures for introduction of MADM cassettes into new loci in the mouse genome, and describe new approaches for expanding the utility of MADM. We show that: 1) Targeted homologous recombination outperforms random transgenesis in generation of reliably expressed MADM cassettes, 2) MADM cassettes in new genomic loci need to be validated for biallelic and ubiquitous expression, 3) Recombination between MADM cassettes on different chromosomes can be used to study reciprocal chromosomal deletions/duplications, and 4) MADM can be modified to permit transgene expression by combining it with a binary expression system. The advances described in this study expand current, and enable new and more versatile applications of MADM.

## Introduction

**Figure 1:**
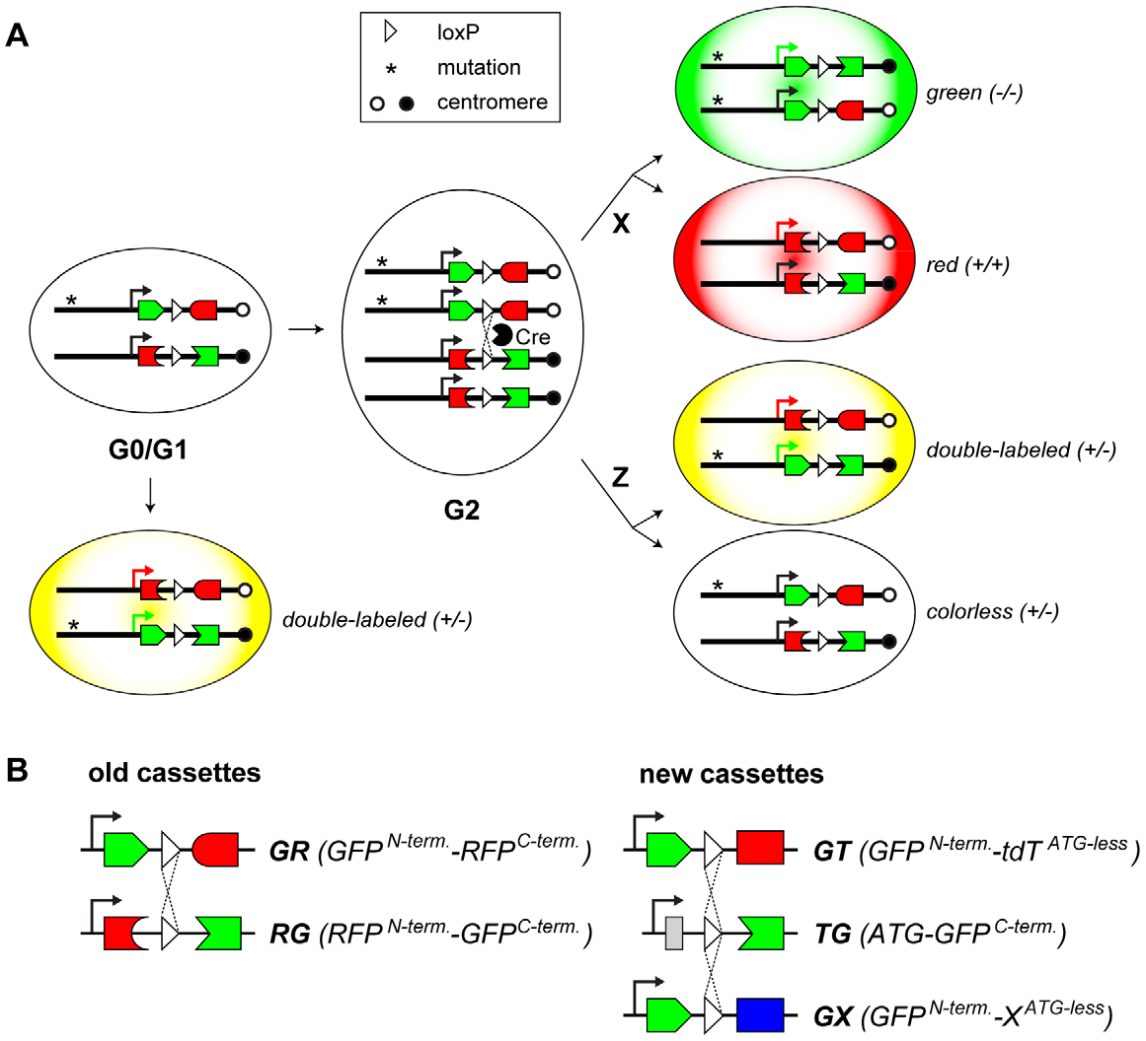
The MADM principle and design of new MADM cassettes. **A**) MADM relies on two reciprocally chimeric marker genes (for example, *GR* and *RG*, see part B below for cassette description) that have been knocked into the same locus on homologous chromosomes. Recombination in the G2 phase of the cell cycle regenerates the functional marker genes on a pair of chromatids. X-segregation of chromatids (the recombinant chromatids segregate to different cells) generates a red and a green cell. Z-segregation of chromatids (the recombinant chromatids congregate to the same cell) generates a double-labeled (yellow) cell and an unlabeled cell. If a mutation (asterisk) is present distally to the *GR* cassette, the green cells will be homozygous for the mutation. This orientation of the cassettes corresponds to MADM in the *Rosa26* locus. If the cassettes are in the opposite orientation with respect to the centromere, the genotypes for green and red cells will be inverted (for example in *MADM-11*). If mitotic recombination occurs in G0 or G1, a double-labeled cell is produced without altering the genotype of the cell. **B**) The “old” MADM cassettes contained two genes encoding fluorescent proteins (dsRed2 and GFP) split roughly in the middle. The “new” cassettes use the same GFP split, but split the second gene (for example, tdTomato) into *ATG* and *Gene^ATG-less^*. That way, the *ATG-G^C-terminus^* (for simplicity, *TG*) becomes a universal cassette that can be paired with any *G-Gene^ATG-less^* cassette. The single white triangle represents a single *loxP* site, a combination of *loxP* sites or the *loxP*-flanked (floxed) neomycin resistance gene (see **Figure S1** for detailed description of MADM cassettes).

Genetically mosaic animals (genetic mosaics) contain cells with different genotypes. Phenotypic analysis of genetic mosaics has become an indispensible tool in modern genetics. Genetic mosaics are usually created by using site-specific recombinases from heterologous biological systems, most prominently including Cre recombinase from the *E. coli* phage P1 [1] and Flp recombinase from the *S. cerevisiae* 2 μ plasmid [2]. DNA recombination can occur either *in cis* (on the same chromosome) or *in trans* (between chromosomes). Intrachromosomal recombination techniques usually rely on the presence of two recombination sites flanking a particular DNA sequence that will be excised upon recombination [3]. In contrast, interchromosomal recombination techniques depend on recombination between chromatids after DNA replication in the G2 phase of the cell cycle to generate sibling cells of different genotypes. Interchromosomal recombination has been used to develop various versions of mosaic analysis in fruit flies [4], [5], [6], [7], [8], [9]. The common and key feature of these approaches is that they create cells with different genotypes *in vivo* and at the same time label those cells with unique markers that strictly correlate with the genotype. To enable such concomitant *in vivo* genetic manipulation and labeling in mammals, we have established Mosaic Analysis with Double Markers (MADM) in mice (**Figure 1A**) [10]. We have used MADM since its inception to perform lineage studies [11] and analyze gene function in a number of biological processes including cell proliferation [12], dendritic patterning [13], neuronal migration [14] and tumor initiation and progression [15]. To expand the utility and versatility of MADM, we present here modifications and new applications of the technique, and compare different procedures for establishment of MADM-ready chromosomes.

## Results

### Design of new MADM cassettes

The original version of MADM relied on the DsRed2 fluorescent protein as one of the two markers [10]. Due to the low DsRed2 fluorescence signal in tests *in vitro*, six Myc epitope tags were added to its C-terminus. The addition of these epitope tags proved to be essential, because the detection of DsRed2 expression from knocked-in MADM cassettes *in vivo* required anti-Myc immunostaining [10]. For the new MADM cassettes, we chose tdTomato (tdT) over DsRed2, due to its improved brightness [16]. We also added three Myc epitope tags to its C-terminus, and this addition did not appear to affect the tdT fluorescence (data not shown).

The original MADM cassettes were designed to split two fluorescent protein genes approximately in the middle of each gene [10] (**Figure 1B**, left). To replace one fluorescent protein gene with another (e.g., DsRed2 with tdT), an entirely new set of cassettes needs to be constructed, as neither of the existing cassettes would be compatible with any new cassette. We therefore aimed to create a more flexible design for new cassettes, such that one of them would be compatible with any new cassette and could be subsequently reused. In our new design for splitting the red fluorescent protein tdT, the first exon contains only the start codon (**Figure 1B**, right). Therefore the two new cassettes are: *GFP^N-terminus^-intron-tdT3Myc^ATG-less^* (for simplicity, *GT*) and *ATG-intron-GFP^C-terminus^* (for simplicity, *TG*). The new *TG* cassette is now compatible with any *GFP^N-terminus^-intron-X^ATG-less^* (for simplicity, *GX*) cassette, where *X^ATG-less^* (for simplicity, *X*) is any gene without the start codon (**Figure 1B**, bottom right). This design has been especially useful for combining MADM with a binary expression system to create MADM-Tet, where X is the tetracycline transactivator tTA2 [17] without the start codon (for details see below).

### Expansion of MADM to additional chromosomes via random transgenesis in ES cells

**Figure 2:**
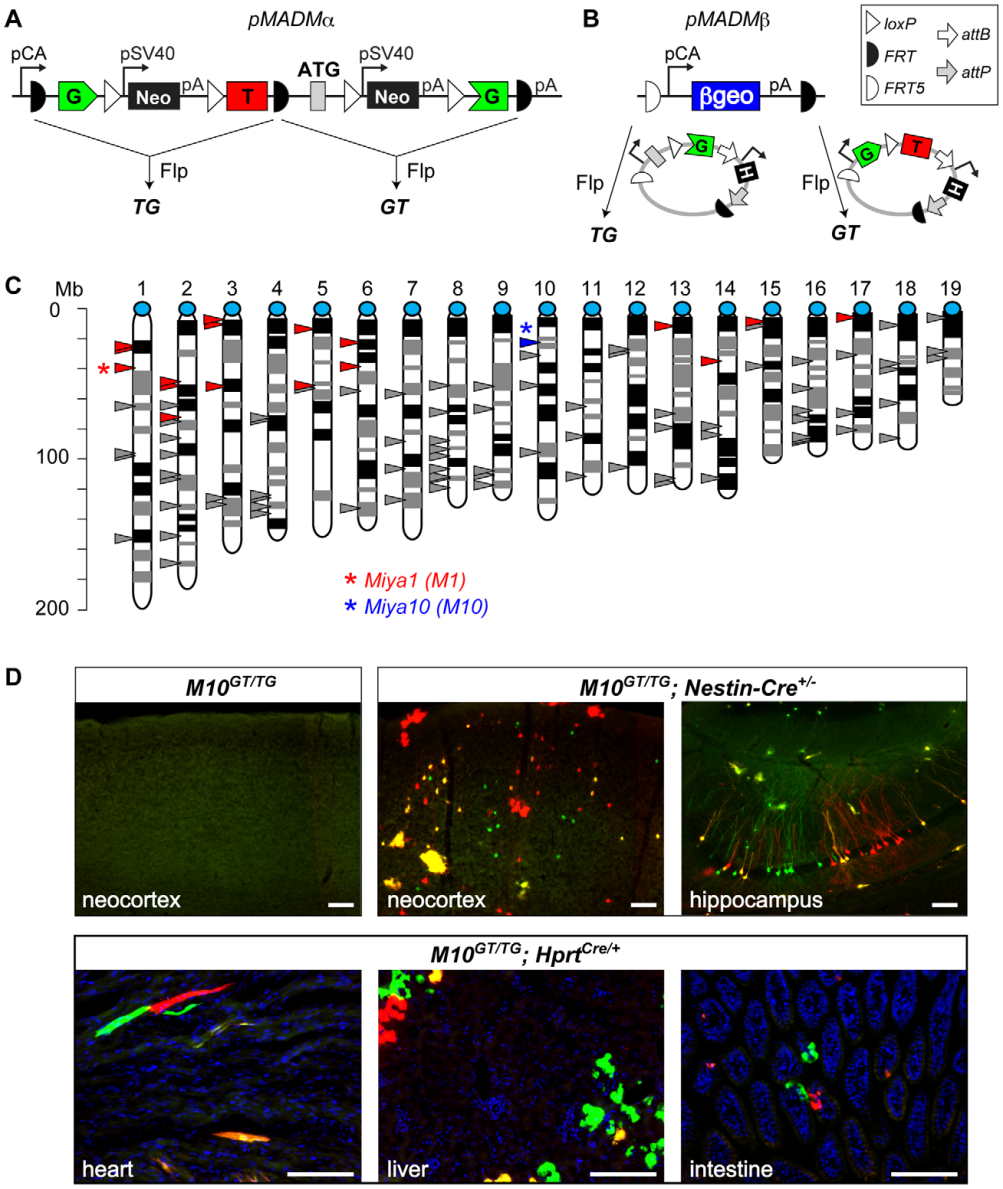
Random integration-based approach to expand MADM to other mouse chromosomes. **A and B**) Schematic representations of MADM precursor (*pMADM*) constructs. **A**) *pMADMα* contains the *CA* promoter, *FRT*-flanked MADM *GT* and *TG* cassettes and a single polyadenylation signal (*pA*). The cassette containing the floxed neomycin phosphotransferase gene (*loxP-pPGK-Neo-pA-loxP*) is placed in the introns of both cassettes. *pMADMα* can be converted into either *GT* or *TG* via partial Flp-mediated recombination in ES cells. **B**) *pMADMβ* construct contains the *CA* promoter driving the *βgeo* gene (a *lacZ* and neomycin-phosphotransferase fusion) flanked by *FRT5* and *FRT*. *pMADMβ* can be converted into any transgene, including a *GT* or *TG* cassette via Flp- and *FRT5/FRT*-mediated cassette exchange in ES cells. These MADM cassettes contained a hygromicin resistance gene (*H*) that was removed by ϕC31 integrase-mediated recombination (see **Figure S1**) before performing the experiments shown in D. **C**) Distribution of *pMADM* transgene *intergenic* integration sites in the mouse genome. Each centromere is represented by a blue circle, and mapped insertion sites are indicated by triangles (Mb, mega base pair). The *pMADMα* insertion site used to establish *MADM-10* (Figure 2D) is represented by the blue triangle located close to the centromere of Chr. 10. All the other triangles represent the insertion sites of *pMADMβ* transgenes based on the 5′ genomic sequence amplified by Splinkerette PCR. Insertion sites that were mapped close to centromeres, and were independently confirmed by both 5′ and 3′ genomic PCR, are represented by red triangles. The insertion located ∼39 Mb from the centromere of Chr. 1 (indicated by an asterisk) was used to establish *MADM-1*. **D**) Representative epifluorescence images of tissue sections with genotypes indicated on top and tissue identity on the bottom. The sections were unstained or stained only with DAPI to label nuclei (blue). The creation of fluorescent cells was Cre-dependent. Scale bars, upper row of images: 100 µm, lower row: 50 µm.

MADM requires two reciprocally chimeric marker genes (‘MADM cassettes’, e.g., *GT* and *TG*), which are targeted into identical loci on homologous chromosomes. When MADM is used to study gene function, the MADM cassettes must be located between the gene of interest and the centromere. This is because only chromosomal segments distal to the recombination sites within the MADM cassettes can undergo exchange and produce homozygosity after X-segregation in the G2 phase of the cell cycle (**Figure 1A**, top right). At present, only genes located on mouse chromosome (Chr.) 6 distal to the *Rosa26* locus and on Chr. 11 distal to the *Hipp11* locus can be subjected to MADM [10], [14]. To extend the MADM technology to other genes in the mouse genome, MADM cassettes need to be inserted into additional chromosomes. One possibility is to employ random transgenesis to obtain integrations throughout the mouse genome. However, random transgene integration of one MADM cassette is in principle not suited for subsequent repeated targeting of the complementary cassette to the same locus. To overcome this problem, we performed random transgenesis using convertible precursor transgenes (*pMADMα* and *pMADMβ*, **Figure 2A, 2B**) that can be subsequently transformed into *GT* and *TG* MADM cassettes.

*pMADMα* contains the ubiquitously active *CA* promoter and *GT* and *TG* MADM cassettes flanked by *FRT* sites. After individual integrants are isolated, they can be converted into *GT* or *TG* cassettes by partial recombination catalyzed by the Flp recombinase (**Figure 2A**). We screened ES cell clones to identify single-copy, intact *pMADMα* transgenes integrated into intergenic regions of the genome (for details see Methods). 25 out of ∼190 ES clones had intact 5′ and 3′ ends of the transgene; 12 of them were estimated to be single-copy based on Southern hybridization; 6 insertion sites were identified by using inverse PCR. Among them, the location of one clone was confirmed to be within an intergenic region, in a new locus we call *Miya10 (M10)*, ∼20 Mb distal to the centromere of Chr. 10 (**Figure 2C**, blue triangle). To obtain *GT* and *TG* transgenes, we introduced the Flp recombinase into this ES cell clone. Among ∼200 ES cell subclones, ten subclones had partial recombination between the second and third *FRTs* to convert *pMADMα* to *GT*, while only one subclone had the reciprocal partial recombination between the first and the second *FRTs* to generate *TG*. We established transgenic mouse lines from these ES cells (hereafter called *M10^TG^* and *M10^GT^*) via standard blastocyst injection to generate chimeras and subsequent germline transmission of the transgenes.

As expected, mice transheterozygous for *GT* and *TG* in the *Miya10* locus (*M10^GT/TG^*) do not have colored cells in the absence of Cre-mediated recombination (**Figure 2D**). When *Nestin-Cre* [18] or *Hprt^Cre^* [19] transgenes were separately introduced to create *M10^GT/TG^;Nestin-Cre^+/−^* or *M10^GT/TG^;Hprt^Cre/+^* or *M10^GT/TG^;Hprt^Cre^/Y*, we observed cells labeled with GFP (green), tdTomato (red), or both (yellow), in patterns predicted by Cre expression. For example, *Nestin-Cre* generates MADM-labeled cells throughout the central nervous system, including cortical pyramidal cells, interneurons, glia, hippocampal granule and pyramidal cells (**Figure 2D**), and cerebellar Purkinje cells (data not shown). *Hprt^Cre^* allows the labeling of cells in the liver, heart, and small intestine (**Figure 2D**) and all other tissues examined (data not shown). When using the same Cre driver, labeling was qualitatively less dense in *MADM-10* than in *MADM-6* or *MADM-11* (data not shown; “*MADM-number*” refers to a genotype, where “*MADM*” signifies two reciprocal cassettes in the same MADM locus combined with a Cre line, while the number refers to a chromosome number). These data demonstrate that random insertion-based transgenesis in ES cells can be used to establish a functional MADM system in a new genomic locus.

However, the *pMADMα*-based approach had two limitations: 1) The frequency of insertion of single copy transgenes was low (∼6%); 2) Partial recombination events to generate *GT* and *TG* cassettes were highly biased in favor of *GT* (10∶1), and therefore obtaining the TG cassette became laborious. These limitations led us to develop a second convertible precursor transgene, *pMADMβ*.

*pMADMβ* contains the *CA* promoter driving the βGeo marker (a fusion of β-galactosidase and neomycin resistance gene), flanked by non-compatible variants of *FRT*: wild-type *FRT* and *FRT5* [20]. This transgene could be subsequently converted into any other transgene, including a *GT* or *TG* cassette via Flp recombinase-mediated cassette exchange [20] (**Figure 2B**). To increase the chance of intact *pMADMβ* integration, ‘protecting’ arms containing bacterial DNA were placed at the 5′ and 3′ ends of the transgene (500 bp and 3.0 kbp, respectively). We electroporated *pMADMβ* into mouse ES cells and isolated ∼1000 subclones; 484 showed strong lacZ expression and 325 (∼32%) had intact 5′ and 3′ ends of the transgene. The intact transgene frequency was ∼2–3 fold higher for *pMADMβ* than for *pMADMα*, presumably due to the longer protection arms. We were able to determine insertion sites for 161 clones using “splinkerette” PCR [21]; 65 insertion sites were located in coding or intronic sequences, and 96 were located in the intergenic areas (triangles in **Figure 2B**). We confirmed the insertion sites by independent genomic PCR for a subset of *pMADMβ*transgene insertions located relatively close to corresponding centromeres (red triangles in **Figure 2B**).

To test the Flp-mediated cassette exchange reaction in ES cells, we selected one single-copy integrant located ∼39 Mb from the centromere of Chr. 1 in the *Miya1 (M1)* locus (**Figure 2B**) and transfected it with a Flp recombinase plasmid and a plasmid containing either the *GT* or *TG* cassette flanked with *FRT5* and *FRT* (see Methods). The cassette exchange efficiency was ∼9% (5 out of 54 sub-clones) or 25% (12 out of 48 sub-clones) for *GT* or *TG* cassettes, respectively. We established mice from these converted ES cells (hereafter called *M1^GT^* and *M1^TG^*) via regular blastocyst injection and chimeragenesis followed by the germline transmission. Similarly to *MADM-10*, we observed Cre-dependent labeling in conjunction with *Nestin-Cre* and *Hprt^Cre^* (data not shown). However, it is important to note that although the cellular labeling obtained by *MADM-1* and *MADM-10* appears as expected (red, green and yellow cells are all evident), this labeling may not accurately report the cellular genotypes unless the loci are biallelically and ubiquitously expressed (see below).

### MADM expansion via targeted knock-in

**Figure 3:**
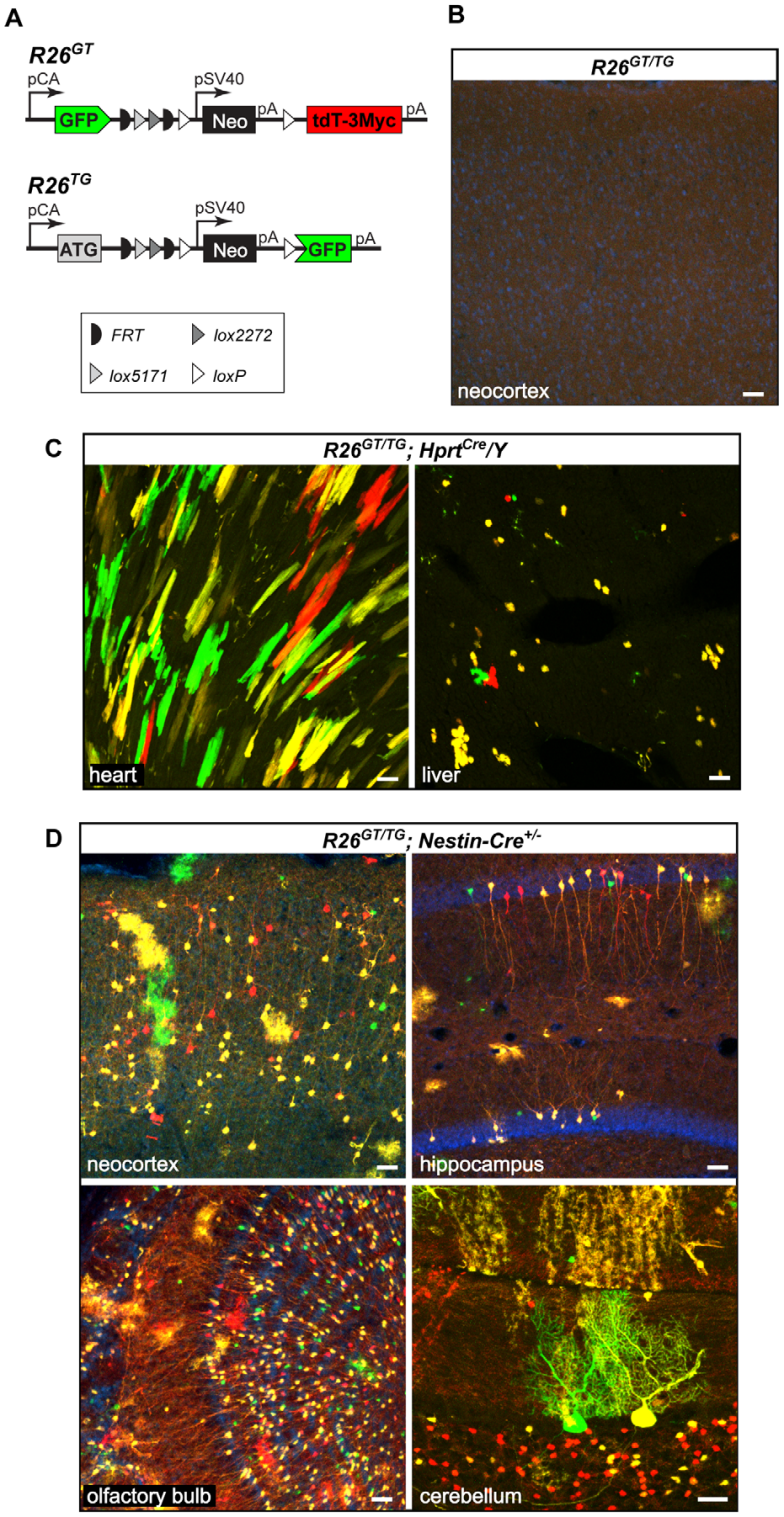
Targeted knock-in approach to create new *Rosa26* MADM with *GT* and *TG* cassettes. **A**) Schematic representation of new alleles: *R26^GT^* and *R26^TG^*. **B), C) and D)** Representative confocal images from tissues indicated on the bottom and genotypes indicated on top. Expected labeling was observed only when Cre was present (compare **B** with **C** and **D**). Bright cellular labeling observed in **C** and **D** originates from native tdT and GFP fluorescence (no additional immunostaining was performed). Some sections were stained with DAPI to label nuclei (blue). Scale bars, 50 µm.

Targeted knock-in [22], [23] is a standard method for introducing a transgene into a precise location in the mouse genome [24]. The vast majority of ubiquitously expressed transgenes, including some made in our lab [10], [25], have been made via knock-in into the *Rosa26* locus [26]. To establish new MADM cassettes (*GT* and *TG*) in a locus that has been already proven to support ubiquitous expression, we inserted them into the *Rosa26* locus. The knock-in procedure into *Rosa26* generated the *GT* and *TG* alleles that allowed marker expression as described previously [10] (**Figure 3**, **S1**). Single-labeled, green and red, cells and double-labeled, yellow, cells were observed only when Cre was present in this version of *MADM-6* containing the new MADM cassettes described above. As expected from *in vitro* cell culture tests, both tdT and GFP fluorescence were visible without immunostaining (**Figure 3**). Thus, this ‘new *MADM-6*’ is superior to the original version of *MADM-6* [10], which required immunostaining to detect the red fluorescent protein.

To expand MADM to other chromosomes via targeted knock-in, we aimed to select loci that should enable the majority of genes on a particular chromosome to be subjected to mosaic analysis, and that are likely to support ubiquitous and biallelic expression of transgenes. Therefore, we focused on chromosomal regions close to the centromere that are located between highly expressed genes as judged by EST abundance [14]. As a specific example, we used the above strategy to knock-in MADM cassettes into the *Hipp11* locus on Chr. 11, and observed Cre-dependent labeling as described [14] (**Figure S1**). We are in the process of generating several other mouse chromosomes with inserted MADM cassettes via the targeted knock-in approach described above.

### Test for biallelic and ubiquitous marker expression

**Figure 4:**
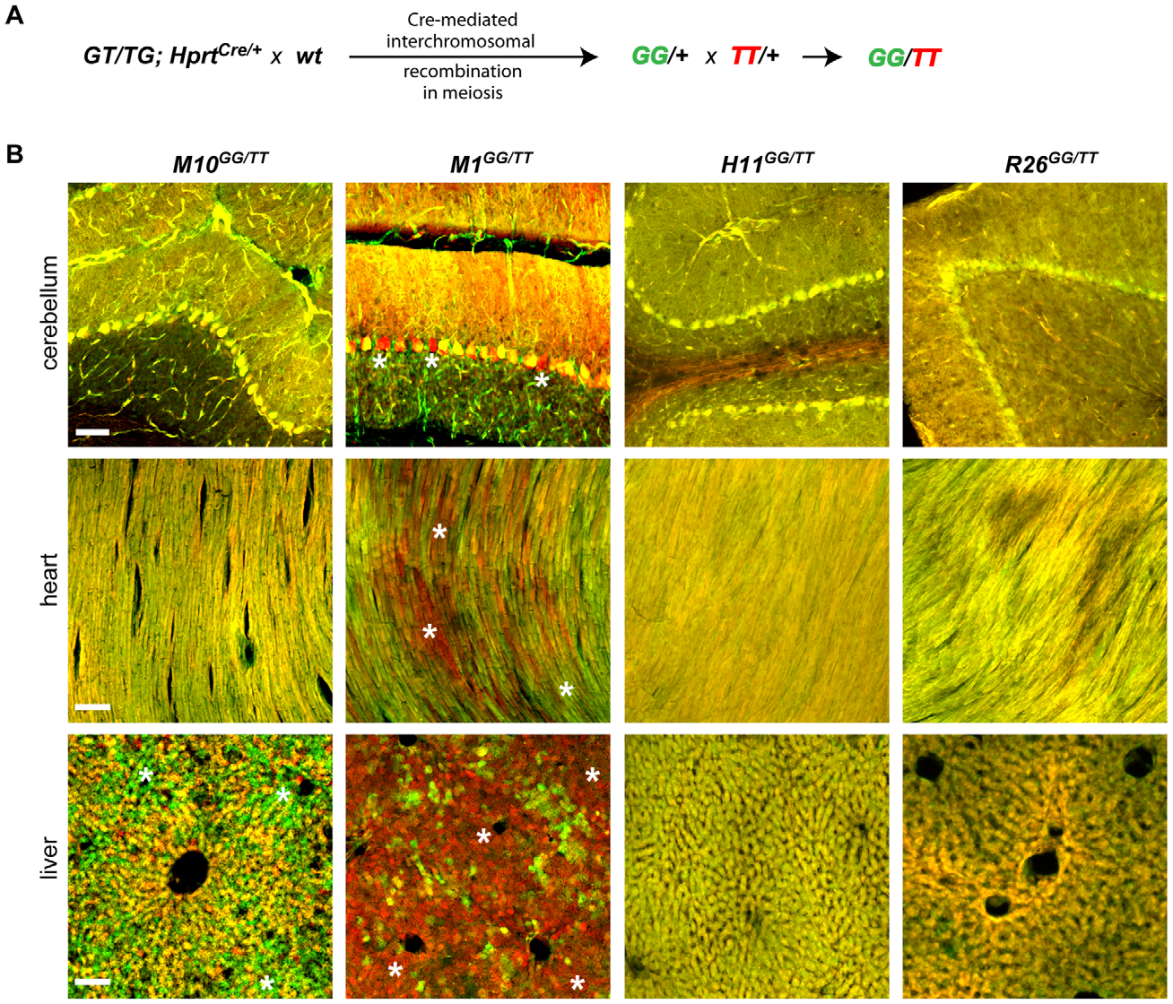
Test for global, biallelic expression from the newly modified MADM loci by creation of *GG/TT* transheterozygotes. **A**) Mating scheme outlines the creation of *GG* and *TT* alleles via Cre-mediated meiotic recombination. The two new lines for each locus were crossed to each other to generate the transheterozygous *GG/TT* animals. **B**) Representative confocal images of unstained tissue sections obtained from animals with genotypes represented above. Cells or groups of cells, in which the expression of one marker is markedly higher than the expression of the other, are indicated by asterisks. Scale bars, 100 µm.

In order for MADM cassettes to reliably report cellular genotypes, the locus containing the cassettes must support biallelic expression in a cellular population of interest. Ideally, the locus should also promote ubiquitous expression. To examine biallelic expression of the new MADM cassettes in all loci examined in this study, we generated the following alleles: *GG* (*GFP^N-terminus^-intron-GFP^C-terminus^*) and *TT* (*tdT3Myc^N-terminus^-intron-tdT3Myc^C-terminus^*). Using the genetic scheme described in **Figure 4A**, we stimulated interchromosomal recombination during meiosis independently in the *Hipp11*, *Miya1*, *Miya10* and *Rosa26* loci to generate *GG* or *TT* alleles in sperm or oocytes. In the process of generation of these alleles, the floxed *Neo* was removed from the introns as confirmed by PCR (data not shown). The newly generated alleles were then transmitted to progeny to generate *GG/+* and *TT/+* animals for all the loci. The frequency of progeny with *GG* or *TT* cassettes was 5.1% (4/78) for *Rosa26*, 15% (4/26) for *Miya1*, 17% (5/29) for *Miya-10*, and 23% (8/35) for *Hipp11*, respectively. In these new transgenes, the expression of GFP or tdT is driven by the CA promoter, which should be ubiquitously active.

To test if a particular locus can indeed support biallelic expression in various organs and cell types, we crossed *GG* and *TT* mice from the same locus and examined labeling in various tissues. If a locus can support biallelic and ubiquitous marker expression, all cells should be double-labeled in an animal of the *GG/TT* genotype. *R26^GG/TT^* and *H11^GG/TT^* appeared to support biallelic and ubiquitous expression in all organs examined (**Figure 4B**). Interpretation of the data for the brain is more complicated due to the structure of neuronal tissue where many different cell types and their processes are intermingled. We therefore focused on the Purkinje cell layer of the cerebellum, where large Purkinje cell bodies can be unambiguously defined. Indeed, all examined Purkinje cells in *R26^GG/TT^* and *H11^GG/TT^* were double-labeled. The same was observed for *M10^GG/TT^* in the cerebellum Purkinje cells and the heart (**Figure 4B**). However, *M10^GG/TT^* in the liver and *M1^GG/TT^* in all examined organs showed highly mosaic expression, displaying many unlabeled or single-labeled cells (**Figure 4B**). Unlabeled cells likely represent biallelic silencing, while single-labeled cells likely represent monoallelic expression/silencing. These data indicate that the *M1* locus does not provide reliable marker expression, and that *MADM-1* cannot be used for phenotypic analysis since it does not guarantee 100% correlation between genotype and marker expression. *MADM-10* can be used in heart and Purkinje cells, but biallelic expression must be tested before application of this system to other cell populations. In summary, the data observed for *MADM-1* and *MADM-10* underscore the importance of testing biallelic marker expression from any new locus harboring MADM cassettes and for any specific cell type before pursuing functional gene analysis.

### Translocations and aneuploidy generated by MADM *in vivo*

**Figure 5:**
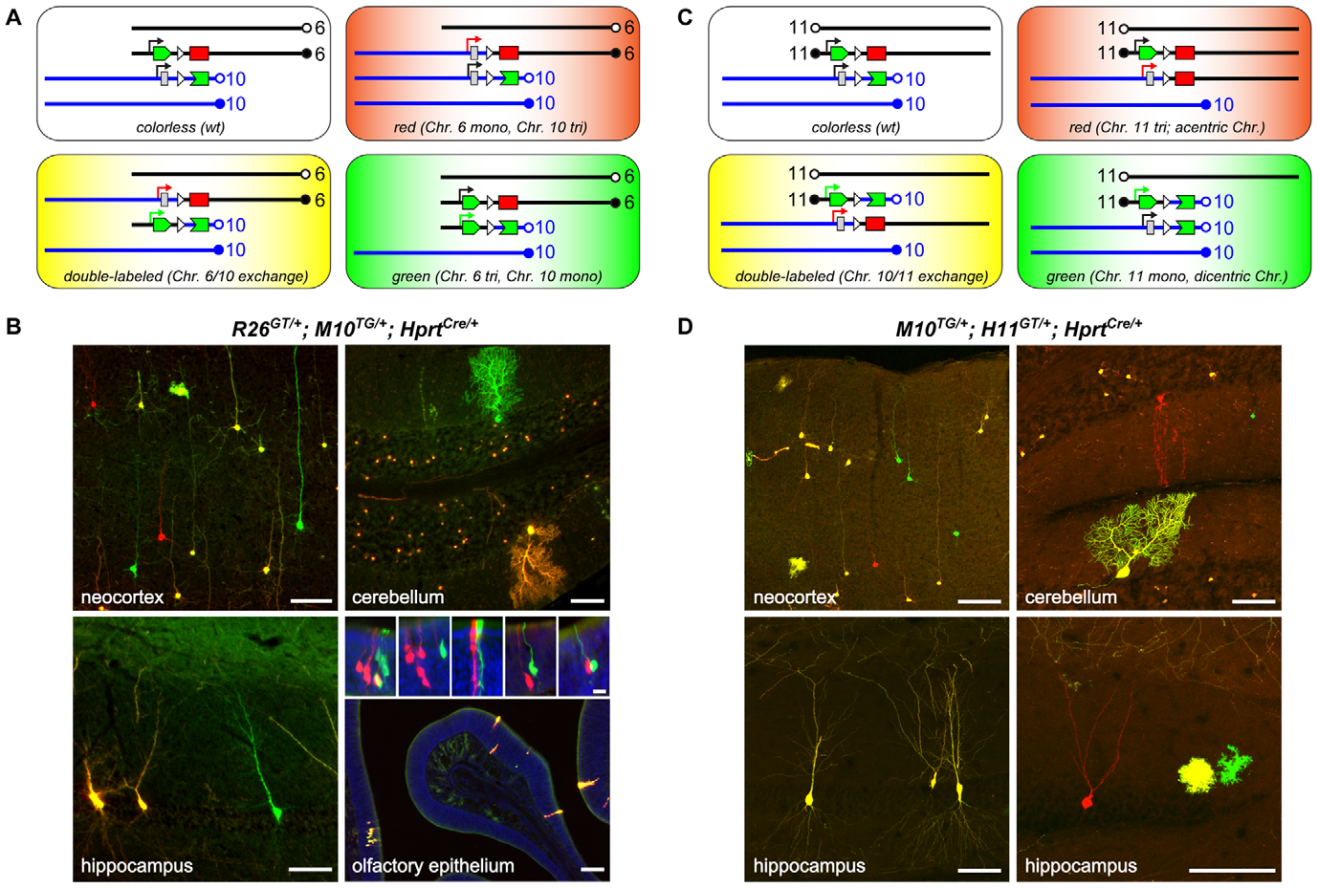
Cells with translocations and aneuploidy generated and labeled by MADM *in vivo*. **A**) Schematic representation of cellular genotypes generated by interchromosomal recombination between non-homologous Chr. 6 and Chr. 10. In both chromosomes, the MADM cassettes are oriented in the telomere-to-centromere fashion. Each double-labeled cell contains the same reciprocal translocation, resulting in no net loss or gain of DNA. Single-labeled (green and red) cells exhibit abnormal copy numbers for parts of the chromosomes distal to the *loxP* sites: red cells are monosomic for the Chr. 6 portion and trisomic for the Chr. 10 portion; green cells have the reciprocal trisomy/monosomy. **B**) Representative confocal images of tissue sections obtained from *R26^GT/+^;M10^TG/+^;Hprt^Cre^/Y* mice. The sections were unstained or stained only with DAPI to label nuclei (blue, in the olfactory epithelium panel). The insets within the olfactory epithelium panel show examples of twin-spot labeling where red and green cells are located in close proximity. Due to the overall low frequency of labeling, each twin-spot labeling most likely originated from a single mitotic recombination event. Scale bars, panels: 100 µm, insets: 25 µm. **C**) Schematic representation of cellular genotypes generated by interchromosomal recombination between non-homologous Chr. 10 and Chr. 11. The MADM cassettes are oriented differently in the two chromosomes with respect to the corresponding centromeres. Each double-labeled cell contains the reciprocal translocation, resulting in one acentric and one dicentric chromosome. Single-labeled cells contain a dicentric or an acentric chromosome, and also exhibit abnormal copy numbers; the red cells are trisomic for Chr. 11 portion distal to *loxP* and monosomic for Chr. 10 portion proximal to *loxP*; the green cells are monosomic for Chr. 11 portion distal to *loxP* and trisomic for Chr. 10 portion proximal to *loxP*. **D**) Representative confocal images of unstained tissue sections obtained from *M10^TG/+^;H11^GT/+^;Hprt^Cre^/Y* mice. Scale bars, 100 µm.

In addition to the interchromosomal recombination between homologous chromosomes described above, MADM could, in principle, mediate interchromosomal recombination between non-homologous chromosomes. These recombination events could generate uniquely labeled cells with reciprocal translocations, a combination of partial trisomy and monosomy, or acentric and dicentric chromosomes (**Figure S2,** **5A, 5C**). Although Cre/*LoxP*-mediated interchromosomal recombination between non-homologous chromosomes was previously demonstrated in mouse ES cells [27], [28] and *in vivo* [29], [30], [31], [32], MADM could permit unambiguous detection and distinction of cells with spatially and/or temporally controlled translocation events by fluorescent markers *in vivo* (**Figure 5A, 5C**).

To test the possibility of recombination between non-homologous chromosomes *in vivo*, we generated transgenic animals containing MADM cassettes on non-homologous chromosomes and a Cre recombinase transgene, and analyzed various tissues for GFP and tdT expression. The genotype and labeling for a particular combination of chromosomes depends on the orientation of MADM cassettes (**Figure 5A, 5C**). It is important to note that in order for these strategies to accurately report cellular genotypes, the loci carrying MADM cassettes need to support their ubiquitous expression (at least at the tissue-wide level). As all our analyses below include the *M10* locus, we performed our tests in the brain, where *M10* appears biallelically and ubiquitously expressed (see **Figure 4B** for the cerebellar Purkinje cells and data not shown).

In animals in which the MADM cassettes are in the same orientation on the centromere-to-telomere axis (e.g., *R26^GT/+^;M10^TG/+^*;*Hprt^Cre/+^*), double-labeled cells should contain a simple reciprocal translocation. Single-labeled (green and red) cells should exhibit abnormal copy numbers for parts of the chromosomes distal to the *loxP* sites. For the particular combination of the *R26* and *M10* transgenes above, the red cells should be monosomic for the Chr. 6 portion and trisomic for the Chr. 10 portion, while the green cells should be monosomic for the Chr. 10 portion and trisomic for the Chr. 6 portion (**Figure 5A**). We observed both single- and double-labeled cells in all tissues examined (nervous system, liver and small intestine, **Figure 5B**). The majority of cells were double-labeled (yellow) suggesting that most labeled cells were generated by post-mitotic recombination (**Figure S2A**) [10]. Another contributing factor to relative abundance of double-labeled cells could be the differential survival of cells with different genotypes. At present, we cannot distinguish between these possibilities.

Interestingly, in the olfactory epithelium, in which extensive post-mitotic cell migration does not occur, we detected “twin-spots” of adjacent green and red cells (**Figure 5B**). Moreover, these single-colored cells were able to further divide several times to form neighboring single-labeled clusters (**Figure 5B** and data not shown). Because the overall labeling frequency was very low, these red and green clusters most likely originated from a single mitotic recombination event. These observations demonstrate that MADM cassettes can be used to create uniquely labeled cells with site-specific reciprocal translocations or aneuploidy *in vivo*. Moreover, we show that cells with this type of aneuploidy are viable and can even divide *in vivo*.

In animals in which the MADM cassettes are in the opposite orientation (e.g., *M10^TG/+^*;*H11^GT/+^;Hprt^Cre/+^*), double-labeled cells should contain an acentric and a dicentric chromosome. Single-labeled cells should contain a dicentric or an acentric chromosome, and also exhibit abnormal copy numbers: red cells should contain an acentric chromosome and should be trisomic for the portion of Chr. 11 distal to *loxP* and monosomic for the portion of Chr. 10 proximal to *loxP*; green cells should contain a dicentric chromosome and be monosomic for the portion of Chr. 11 distal to *loxP* and trisomic for the portion of Chr. 10 proximal to *loxP* (**Figure 5C**). We observed both single- and double-labeled cells in various parts of the nervous system (**Figure 5D**). Again, most of the cells were double-labeled, and may have arisen postmitotically. It is important to note that in this case, the recombinant chromosomes that produce labeling are either dicentric or acentric. Unreliable transmission and loss of dicentric and acentric chromosomes in mitosis may therefore result in “conversion” of double-labeled cells to single- or unlabeled cells. Equivalent loss of acentric or dicentric chomosomes could convert single-labeled cells into unlabeled cells. As double-labeled cells can be generated both mitotically and postmitotically (**Figure S2B**), while the single-labeled cells can be generated only mitotically, the numbers of single-labeled cells may be disproportionately decreased compared to double-labeled cells due to the loss of dicentric and acentric chromosomes during mitosis.

### MADM-Tet: combining MADM with a binary expression system

Introduction of a binary expression system into MADM could expand the scope and utility of the technique by permitting expression of any transgene in one of the two mitotically generated and uniquely labeled sibling cells. In addition, if a binary system can be regulated, it would enable new types of analyses and enhance their spatial and temporal resolution. These additional capabilities could be used to: 1) rescue mutations with transgenes and test the critical periods of gene function (mutation in gene X combined with temporally-regulated expression of gene X); 2) test genetic interactions (mutation in gene X and temporally-regulated expression of gene Y, or dominant negative gene Y); 3) test the effect of gene overexpression; or 4) enable versatile subcellular labeling (e.g., synapse-specific labeling by expressing Synaptophysin-GFP fusion protein to assess synaptic phenotypes) [33]. For example, the inclusion of one or more binary expression systems in the similar mosaic system in flies (Mosaic Analysis with a Repressible Cell Marker, MARCM) [6] has greatly extended its utility [9], [34].

**Figure 6:**
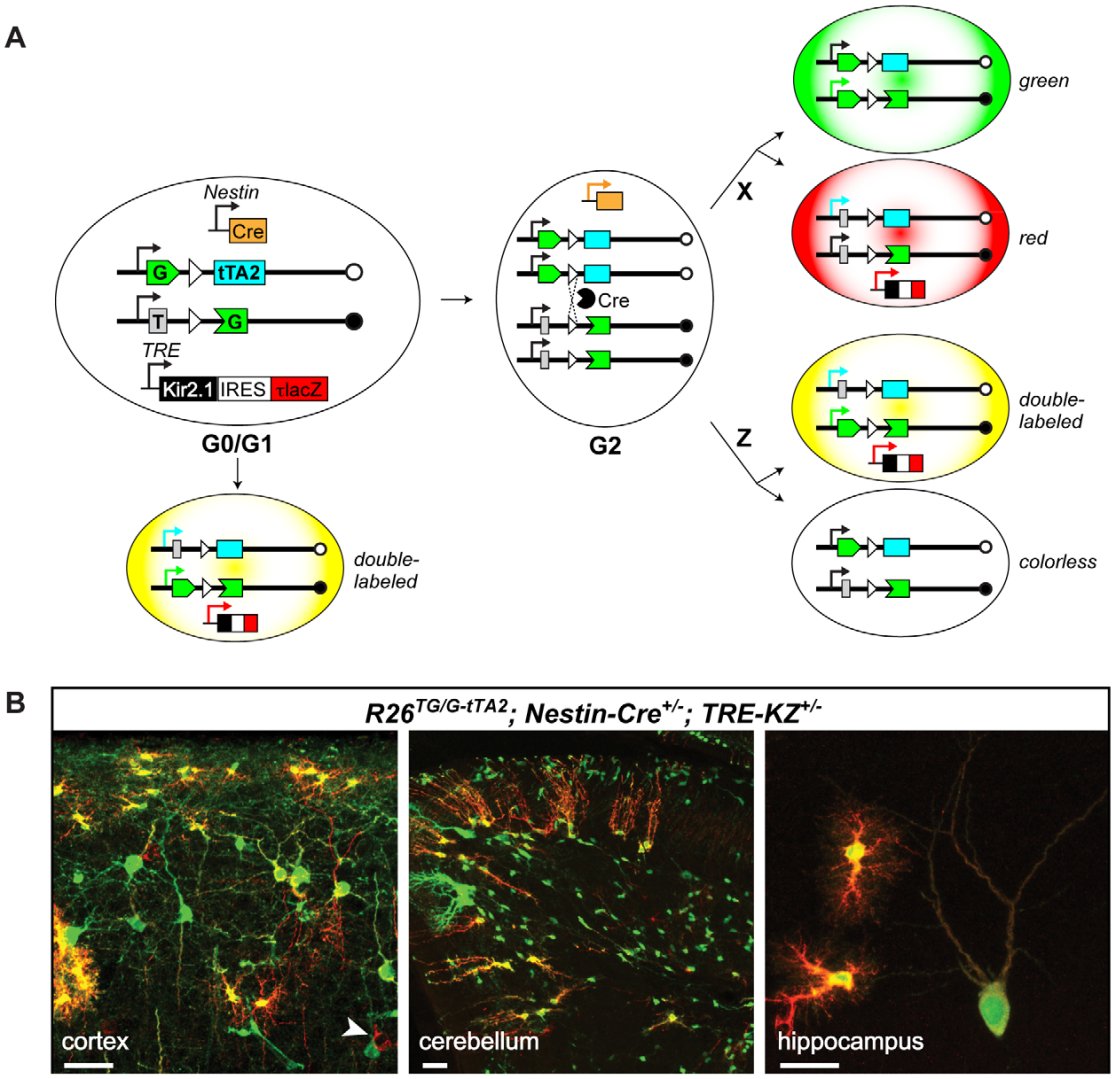
MADM-Tet combines MADM with a binary expression system. **A**) Schematic representation of MADM-Tet starting with the following genotype: *R26^TG/G-tTA2^;Nestin-Cre^+/−^;TRE-KZ^+/−^*. Although all cells contain the *Nestin-Cre* and *TRE-KZ* transgenes, for simplicity they are displayed within the cells only when they are active. **B**) Confocal images of tissue sections stained with antibodies against GFP (green) and lacZ (red) from mice of the genotype indicated above. Note that the two markers exhibit different subcellular distribution: GFP labels whole cells including nuclei, whereas tau-lacZ is absent from the nuclei and labels the processes more strongly (an example of a red-only cell body is indicated by an arrowhead). Scale bars, left and middle panels: 50 µm, right panel: 25 µm.

The new design of MADM cassettes allowed us to reuse the *TG* cassette for this purpose. In addition, we generated another new cassette containing a split transcription factor, the tetracycline transactivator, tTA2 [17]. Therefore, the two new cassettes for MADM-Tet are: *ATG-intron-GFP^C-terminus^* (*TG*) and *GFP^N-terminus^-intron-tTA2^ATG-less^* (*G-tTA2*). The plasmids containing these cassettes were tested in tissue culture to show that they express functional GFP and tTA2 only in the presence of Cre (data not shown). We knocked-in the new *G-tTA2* cassette into the *Rosa26* locus, and tested it by creating a quadruple-transgenic mouse: *R26^TG/G-tTA2^*;*Nestin-Cre* ^+/−^;*TRE-KZ^+/−^* (**Figure 6A**). *TRE-KZ*, (originally called *tet_o_-Kir2.1-IRES-tau-LacZ*), is a random transgene encoding the potassium channel Kir2.1 and a tau-LacZ fusion under the control of the TRE promoter [35]. Immunostaining against GFP and LacZ revealed the two antigens: the GFP signal (green) was distributed throughout the cell, while the tau-LacZ signal (red) was predominantly located in neuronal and glial processes (**Figure 6B**). Thus, MADM-Tet enables a TRE transgene to be expressed in a subset of MADM-labeled cells.

## Discussion

MADM is a powerful tool for high-resolution mosaic analysis of gene function in mice *in vivo* that requires MADM cassettes on the chromosomes harboring genes of interest [10], [12], [13], [14], [15]. Therefore, to maximize the applicability of MADM to most genes in the mouse genome, it would be ideal to establish MADM cassettes near the centromeres of all mouse autosomes.

Here, we created new mice that harbor MADM transgenes on different chromosomes, and compared two different methods for introducing MADM cassettes into new loci. We also established a test for biallelic and ubiquitous marker expression from these new loci harboring MADM transgenes. Using this test, we observed ubiquitous and biallelic expression from two targeted knock-ins, but stochastic expression from two randomly introduced transgenes. At present, we cannot determine whether the stochastic expression of the single-copy, randomly integrated transgenes is due to the loci themselves or due to the fact that all randomly integrated transgenes characterized in this study also contain plasmid bacterial DNA, which was used as a “buffer” to protect transgene ends. Our recent observations during the development of a site-specific transgenesis technique show that bacterial DNA can have a severe silencing effect, which is most prominent in the liver [36]. The silencing effect of bacterial elements on mammalian transgenes has been observed before in randomly integrated transgenes [37] and episomal transgenes [38]. This correlation suggests that the bacterial sequences flanking these transgenes could contribute to their variable expression. Therefore, targeted knock-ins or new random transgenesis screens, where bacterial protection arms are avoided, should be the methods of choice for expanding MADM cassettes onto other chromosomes.

In the future, we recommend that new genomic loci harboring MADM cassettes should be tested for biallelic expression by creation of *GG* and *TT* alleles from *GT* and *TG* alleles (**Figure 4A**). To expedite this key validation experiment, we recommend creation of the GG allele before or in parallel to the *GT* and *TG* alleles. The ubiquity of the expression in the new locus can then be tested by examining the *GG* allele alone [10] or by crossing it to *R26^TT^* and assessing the extent of double-labeled cells for any cell type of interest. However, the most rigorous test for biallelic expression should finally be performed by crossing the *GG* and *TT* alleles in the same locus as described in **Figure 4**.

Visual inspection of the efficiencies of MADM labeling (including both single- and double-labeled cells) revealed that *H11*>*R26*∼*M1*>*M10*. For *M10* and particularly *M1*, these estimates are not completely reliable as these loci are not reliably expressed. Nevertheless, these data suggest that homologous recombination efficiencies differ quite widely for different chromosomal loci in somatic cells *in vivo*, and they are consistent with similar findings previously reported in embryonic stem cells [39]. The expression levels of the marker genes driven by the same *pCA* promoter in *M10*, *H11* and *R26* loci do not appear dramatically different, at least in tissues in which the markers are reliably expressed.

We also show that complementary MADM cassettes on different chromosomes can be used to produce and label cells that undergo various translocation events. This new application of MADM now permits the analysis of single-cell phenotypes produced by precisely defined translocation events *in vivo*. Interestingly, in the *M10/H11* translocation case, we observed double-labeled Purkinje cells with elaborate dendritic trees, suggesting that the presence of dicentric and acentric chromosomes does not perturb the development or maintenance of a complex dendritic arbor. Systematic studies in the future can determine the consequences of chromosomal aneuploidy on the differentiation and function of different cell types.

Finally, we demonstrate that by replacing one of the fluorescent markers with the tTA2 transcription factor, MADM can also express a TRE-controlled transgene of interest in a small population of cells. This capability can be used in the future to combine transgene expression with loss-of-function mutations in the same, uniquely labeled cells. Further modifications of the technique would extend MADM-Tet capabilities. For example, a new G-tTA2 cassette that would include labeling of tTA2-expressing cells independently of the tTA2 activity would allow their visualization before or after the expression of tTA-dependent transgenes. Efficient generation of reliable TRE transgenes would further facilitate the use of MADM-Tet. As a built-in *TRE* transgene was mostly silent in the *Rosa26* locus (as part of the *G-TET* allele; **Figure S1B** and **S3**) and because TRE transgenes are prone to silencing [40], [41], we have been modifying the *TIGRE T1* [42] locus to enable integrase-mediated site-specific transgenesis [36] for efficient creation of reliable TRE transgenes. Together, these advances enable new applications of MADM and will facilitate additional extensions of MADM in the future.

## Methods

### Ethics Statement

All animal procedures were in compliance with the institutional animal care guidelines and were approved by Stanford University's Administrative Panel on Laboratory Animal Care (A-PLAC, protocol number 14007).

### Plasmid construction

Recombinant DNA was constructed using standard techniques. When fragments were amplified by PCR, we used Phusion Taq polymerase (Finnzymes), and confirmed the sequences fully by DNA sequencing. All synthetic DNA fragments were also fully confirmed by DNA sequencing.

***pMADMα (pCA-FRT-G-Neo-T-FRT-T-Neo-G-FRT-pA).*** *pCA* promoter (containing the chicken β-actin promoter and a CMV enhancer) and the SV40 polyadenylation signal (*pA*) from *pCA (HZ2)* [10], and synthetic DNA fragments containing *FRT* sites were sequentially introduced into pBluescript to create a plasmid intermediate, *pKM3* (*pCA-FRT-XmaI-EcoRI-FRT-SpeI-HindIII-FRT-pA*). The *XmaI/EcoRI* fragment of MADM-*TG* cassette [14] (see construction details below) was introduced into a pBluescript vector to flank this cassette with *SpeI* and *HindIII*. Then, the *SpeI/HindIII* restriction fragment of MADM-*TG* cassette, and *XmaI/EcoRI* fragment of MADM-*GT* cassette [14] were sequentially introduced into *pKM3* to generate *pMADMα*. The construct was digested with restriction enzymes PvuI and AflIII, and the insert was gel-purified using Qiagen gel extraction kit and eluted into 10 mM Tris-HCl, pH 7.4, 0.1 mM EDTA. The purified and linearized DNA contained ∼50 bp and ∼300 bp of vector sequence at its 5′ and 3′ ends, respectively. This vector sequence was deliberately retained to minimize the transgene damage with exonucleases after electroporation of the DNA into mouse ES cells.

***pMADMβ (pFRT5-pCA-βGeo-pA-pPGK-TK-pA-FRT).*** The following fragments were assembled together in this order to make *pMADMβ*:

- 5′ protection arm: ∼500 bp PCR fragment of *β*-*lactamase* gene from pBluescript amplified by the following primers: GGTACCATTTAAATAGATTATCAAAAAGGATCTTCACC and GGTACCTAACTCGCCTTGATCGTTGG.
- 319 bp PCR fragment of *wheat germ agglutinin* (*WGA*) gene amplified by PCR primers: GGTACCGACGTGTCCCAACAACCACT and AAGCTTCATGCCACAGGATCCCCACT. This arm was placed immediately downstream of the protection arm to provide unique sequence in the mouse genome for the Splinkerette PCR. Single *NlaIII* restriction site was artificially introduced in the 3′ end of this arm for Splinkerette PCR.
- *FRT5* (GAAGTTCCTATTCCGAAGTTCCTATTCTTCAAAAGGTATAGGAACTTC) from a synthetic DNA was introduced after the unique 5′ arm.
- *pCA* promoter from *pCA (HZ2)* [10].
- *SalI/KpnI* fragment containing the *βGeo* gene from the plasmid *Z/EG* [43].
- *KpnI/HindIII* fragment containing the *pPGK-TK-pA* cassette from the plasmid *pLOXPNT* [24]. (Note that we eventually decided not to use thymidine kinase (TK)-based selection.)
- *FRT* (GAAGTTCCTATTCCGAAGTTCCTATTCTCTAGAAAGTATAGGAACTTC) from a synthetic DNA was introduced after the TK cassette.
- 218 bp PCR fragment of yeast *His3* gene amplified by PCR primers GCGGCCGCTCGAAGTAGCCGCCGTTGTTGTTAT and GAGCTCGGTGATAGGTGGCAAGTGGT. (Note: This sequence was originally introduced as a 3′ unique area for screening purposes, but was not used in this study.)

The construct was digested with restriction enzymes PvuI and AflIII, and the insert was gel-purified using Qiagen gel extraction kit and eluted into 10 mM Tris-HCl, pH 7.4, 0.1 mM EDTA. The purified and linearized DNA contained ∼500 bp and ∼3000 bp of vector sequence at its 5′ and 3′ ends, respectively. The vector sequences served as protection arms against endonucleases after electroporation into the mouse ES cells.

***pExG*** and ***pExT*** *(pFRT5-pCA-GT-pA-FRT-attPx3-pPGK-Hyg-pA-attB* and *pFRT5-pCA-TG-pA-FRT-attPx3-pPGK-Hyg-pA-attB)*: Synthetic DNA fragments containing *FRT5* [20], [44] and *FRT* were sequentially introduced 5′ and 3′ to the MADM cassettes in the MADM-*GT* and *TG* constructs to generate intermediates ***pFRT5-GT-FRT*** and ***pFRT5-TG-FRT***. Independently, ***pattB-Hyg-attPx3*** was generated by flanking the hygromycin resistance gene (*Hyg*) driven by the phosphoglyceratekinase promoter (*pPGK*) with ϕC31 integrase recognition sites: three 70-bp long *attP* sites from *pBT298* [36] and a “full length” *attB* [45]. To create the final constructs (*pExG* and *pExT*), the *XmaI/BamHI* fragment from *pattB-Hyg-attPx3* was introduced into the *SwaI* site of *pFRT5-GT-FRT* and *pFRT5-TG-FRT*. The constructs were prepared by using Endotoxin Free-Maxi prep (Qiagen) for the electroporation into the mouse ES cells.

### MADM cassettes

To construct and test final MADM targeting constructs we created a set of constructs in the *pCA (HZ2)* plasmid, which contains a polylinker between the *pCA* promoter (chicken β-actin promoter and CMV enhancer) and an *SV40* polyadenylation site [10].

***pCA-G-intron-T.*** The previously used first *GFP* exon from the *GR* cassette [10] and *tdT3Myc^ATG-less^* were assembled in *pHZ2* separated by the previously described modified *β-globin intron* containing *BglII* and *loxP* sites [10].

***pCA-T-intron-G.*** The previously used second GFP exon from the *RG* cassette [10] was assembled with a fragment containing a Kozak sequence, ATG start codon and the same *β-globin intron* desribed above in *pHZ2*.

***pCA-G-intron^Neo^-T*** **and** ***pCA-T-intron^Neo^-G*** **.** We inserted a *BglII/BamHI* fragment containing the neomycin resistance gene (*Neo*) driven by an *SV40* promoter and followed by the *HSV TK* polyadenylation site into the *BglII* site of *pCA-G-intron-T* or *pCA-T-intron-G*, respectively. We created three different versions of the *Neo* cassette to contain different numbers or identities of recombination sites: *version 1:* ***pLN*** *: loxP-pSV40-Neo-pA; version 2:* ***pFLN*** *: FRT-loxP-pSV40-Neo-pA; version 3:* ***pFLLFLN*** *: FRT-Lox5171-Lox2272-FRT-loxP-pSV40-Neo-pA*. The *loxP* versions, *Lox5171* and *Lox2272* are incompatible with each other and with *loxP*, but each one is compatible with itself [46]. They were introduced in attempts to increase recombination efficiency. Comparisons of these intron versions and their effect on recombination efficiency will be described elsewhere (A. Henner and H. Zong, in preparation). The intron versions that were used for creating targeting constructs for particular loci described in this study are schematically represented in **Figure S1**.

***pBT234 (pCA-G-intron-tTA2^ATG-less^-pA).*** Used for testing the cassettes before the construction of final targeting constructs.

***pBT250 (pCA-G-intron^Neo^-tTA2^ATG-less^-pA).*** *pGLLFNL* was cloned into *BglII* site of *pBT234*.

***pBT270*** **(** ***pCA-G-intron^Neo^-tTA2^ATG-less^-pA-ii-TRE-tdT3Myc-pA-ii).*** *ii-TRE-tdT3Myc-pA-ii* from *pBT264* (*pii-TRE-tdT3Myc-pA-ii*) [47] was inserted into *pBT250*.

### Targeting constructs

All targeting constructs for the *Rosa26* locus were created by inserting a *PmeI/AscI*-digested fragment from a precursor plasmid into the *pROSA26-PA* [48]. The ***pRosa26-GT*** precursor is *pCA-G-intron^Neo^-T*; ***pRosa26-TG*** precursor is *pCA-T-intron^Neo^-G;* ***pRosa26-G-tTA2 (pBT259)*** precursor is *pBT250*; ***pRosa26-GTET (pBT272)*** prescursor is *pBT270*.

### Control constructs

***pBT255 (pCA-GFP4m)*** **.** Used as a positive control for GFP expression. GFP4m (or mut4EGFP) is a thermotolerant GFP variant [49], [50]. All other GFP-containing constructs in this study contain this variant of GFP.

***pCA-G-intron-G***. Described originally in [10]. Used as a positive control for split GFP expression.

***pBT225*** *(pCA-tdT3Myc)*: Used as a positive control for tdT expression and Myc staining.

***pCA-ATG-intron-tdT***. Constructed initially as a test for splitting the *tdT* gene into an exon containing a start codon (*ATG*) and and an exon containing the rest of tdT by the *β-globin intron* containing the *BglII* site and *loxP* [10].

***pBT224 (pCA-tTA2-pA)*** **.** Used as a positive control for tTA2 activity in conjunction with *pBT239* (*TRE-tdT3Myc-pA*) [47]. *tTA2* gene was subcloned from *pUHT61-1* [17].

***pBT241 (pCA-ATG-intron-tTA2^ATG-less^).*** Constructed initially as a test for splitting the *tTA2* gene into *ATG* and the rest of *tTA2* by the *β-globin intron*. PCR was used to construct two DNA fragments: *XmaI-ATG-intron* and *tTA2^ATGless^-EcoRI*. The *β-globin intron* was modified at the same position as described previously [10] to contain single *BglII*, *loxP* and *FRT* sites. The two fragments were ligated to each other (via blunt ends) and to *BglII/EcoRI*-digested *pCA (HZ2)* [10] in a 3-way ligation reaction. This construct was used to test the functionality of tTA2 after insertion of the intron in conjunction with *pBT239* (see above).

***pBT267*** **(** ***pCA-ATG-intron-tTA2^ATG-less^-TRE-tdT3Myc-pA)*** **and** ***pBT268*** **(** ***pCA-ATG-intron-tTA2^ATG-less^-iiTRE-tdT3Myc-pAii*** **).** Constructed to compare the effect of insulators on decreasing tTA2-independent activation of *TRE*. The constructs were also tested in the presence of doxycycline to assess which construct has higher background expression of tdT3Myc.

### Screening of ES cell clones obtained by random transgenesis

We used standard techniques [24] to modify R1 mouse ES cells, which originated from a 129 mouse strain [51].

*pMADMα* construct was introduced into ES cells via electroporation, and individual G418-resistant clones were evaluated for intact transgene integration by genomic PCR using primers KM1 (GTGCTGCAAGGCGATTAAGT) and KM2 (TTATGTAACGCGGAACTCCA) to detect the 5′ end of each transgene (PCR product, 211 bp), and CCCCCTGAACCTGAAACATA and TGTGGAATTGTGAGCGGATA to detect the 3′ end of each transgene (PCR product, 275 bp). We further analysed the genomic DNA from the ES cells containing intact transgenes by Southern blotting. We used a probe for *Neo*, which is located in the intron of MADM cassette, and the genomic DNA obtained from the *R26^TG/+^* mouse line as a reference for a single-copy transgene. For ES cells that contained single-copy transgenes based on the Southern blot, we performed inverse PCR to identify the 5′-flanking genome sequence. Genomic DNA was digested with restriction enzyme NlaIII and subjected to the ligase-mediated self-ligation. The resultant circular DNA was then used as a template for a two-step nested PCR to amplify the transgene flanking region. For the first round of PCR, we used primers: TAATCGAAACCCTGGCGTTA and GTTTTCCCAGTCACGACGTT. For the second round of PCR, we used 0.3 µl of the first round PCR product and primers: ACTTAATCGCCTTGCAGCAC and TATAGGGCGAATTGGGGAAT. The final PCR products were analyzed by electrophoresis, gel-extracted by the Qiagen gel extraction kit, and analyzed by DNA sequencing. For the intact single-copy transgenes integrated in intergenic regions close to any centromere, we performed additional Splikerette PCR [21] to confirm integration sites.

*pMADMβ* was also introduced into R1 mouse ES cells by electroporation and individual G418-resistant clones in 96-well plates were evaluated for the expression of βgeo by lacZ staining. 96-well plates were washed with PBS, fixed by 0.2% glutaraldehyde in PBS for 5 min. at room temperature (RT), and washed 3 times at RT with the staining buffer (2 mM MgCl2, 0.01% Deoxycholate, 0.02% NP40, 100 mM phosphate buffer, pH 7.5). Cells were then treated with the solution containing: 5 mM potassium ferricyanide (Sigma), 5 mM potassium ferrocyanide (Sigma), 2.5 mM 5-bromo-4-chloro-3-indolyl-beta-D-galactopyranoside (X-gal, Invitrogen) at 37°C for 1-2 hours. We recorded the activity of lacZ in individual clones and then extracted genomic DNA from 96-well plates. We tested the intactness of 5′ and 3′ ends of the transgenes by PCR. We used the following primers: CTATGCCCGAACAACCTCTG and ATCATATGCCAAGTACGCCC (for *pMADMβ* 5′ end); CCCCCTGAACCTGAAACATA and ACCACTTGCCACCTATCACC (for *pMADMβ* 3′ end). The clones with high lacZ expression and intact transgenes, were further analyzed by Splinkerette PCR [21] to identify the flanking genomic sequence at the 5′ end of the transgene. Splinkerette PCR was reported to be more efficient than inverse PCR method we used for *pMADMα* screening. The genomic DNA was digested with NlaIII and a specifically designed Splinker was ligated overnight at 16°C. To make the Splinker adaptor, two synthetic DNA oligonucleotides: TAACCGTTGCTAGGAGAGACCGTGGCTGAATGAGACTGGTGTCGACACTAGTGGCATG and CCACTAGTGTCGACACCAGTCTCTAATTTTTTTTTTCAAAAAAA were annealed. The ligation products were purified by the Qiagen PCR purification kit and eluted into 30 ml of TE. 1 µl of the sample was used for the first round of PCR with primers: AACCGTTGCTAGGAGAGACC and CCGCAGAACTCGGAACCTA. 0.3 µl of the first PCR product was subjected to the second round of PCR with primers: GCTGAATGAGACTGGTGTCG and CAGAGGTTGTTCGGGCATAG. The final PCR products were analyzed by electrophoresis, gel-extracted by the Qiagen gel extraction kit, and analyzed by DNA sequencing.

### Converting *pMADM* transgenes into *GT* or *TG* cassettes

To convert *pMADMα* to MADM-*GT* or *TG*, 5 µg of a plasmid (***pPGK-Flpo-Puro***) containing a codon-optimized Flp recombinase gene (*Flpo*) [52] and the *puromycine resistance gene* was introduced into 5 million cells of selected clones via electroporation. We cultured ES cells with 200 µg/ml G418 and 2 µg/ml puromycine for 48 hours, and ES cell were subsequently cultured only in the presence of G418 until colonies formed. Individual G418-resistant clones were analyzed by two genomic PCRs to specifically detect 5′ and 3′ parts of *pMADMα* and identify non-recombined clones and partially recombined clones (*GT* or *TG* cassette). One PCR used primers GCAACGTGCTGGTTATTGTG and CGCCTCAGGACTCTTCCTTT to amplify ∼600 bp product for non-recombined *pMADMα* cassette and MADM-*GT* cassette or ∼300 bp product for MADM-*TG* cassette. Another PCR used primers GAAACTGGGCATGTGGAGAC and TGAGTTTGGACAAACCACAAC to amplify ∼800 bp product for non-recombined *pMADMα* cassette and MADM-*TG* cassette or ∼2.0 kb PCR product for MADM-*GT* cassette. The ES cell clones that contained correctly recombined cassettes were used to generate chimeric mice by injection into C57BL/6 blastocysts.

To convert *pMADMβ'1* into MADM-*GT* or *TG*, 25 mg of one of the exchange cassette plasmids (*ExG* or *ExT*) and 25 mg of *pPGKFLPobpA* plasmid (Addgene plasmid 13793) [52] were introduced into the selected ES cells (∼5×10^6^ cells). We cultured the ES cells without selection for 72 hours and then applied hygromycin (120 mg/ml) for one week. Individual hygromycin-resistant clones in 96-well plates were divided into five replicas: two for stock, one for lacZ staining, and two for genomic DNA preparation. To detect the *FRT5*/*FRT* mediated site-specific recombination events, we used PCR with primers: CTATGCCCGAACAACCTCTG and GGGCGTACTTGGCATATGAT to amplify the junction containing *FRT5*. This PCR not only confirmed that the ES clone still contained the MADM transgene, but also generated different sizes of PCR products for non-recombined (510 bp) and recombined (550 bp) clones. The PCR products were analyzed by electrophoresis on a 2% agarose gel. Site-specific recombination was confirmed by additional PCR primer sets that specifically amplify the newly formed junctions at *FRT5* (5′ of the transgene): CTATGCCCGAACAACCTCTG and TCCCAGTCCTTGCCATTTAG (create a 286 bp product) and *FRT* (3′ of the transgene): AAGCATCAACGACAACAACG and 5′- CGGAATACCACTGAAATTGG (create a 200 bp product). The ES cell clones that contained correctly recombined cassettes were used to generate chimeric mice by injection into C57BL/6 blastocysts. The chimeras were directly crossed to a ϕC31o integrase mouse line (Jackson laboratory, stock# 007670) [52] to remove the *pPGK-Hyg-pA* cassette from the genome in the next generation.

### Tissue processing, immunohistochemistry and imaging

Tissues were processed according to previously described procedures [10], [11]. Neither tdT nor GFP required immunostaining for visualization. Although the majority of the data presented in the paper were obtained from unstained tissue sections, sections can be immunostained for better signal preservation according to previously published methods [11] using the following primary antibodies: chicken anti-GFP (1∶500; Aves Labs), goat anti-MYC (1∶200; Novus; the best results are obtained if antibody is pre-absorbed with fixed, finely minced, wild-type brain according to the previously described procedure [33]), rabbit anti-DsRed (1∶1000; Clontech), or rabbit anti-LacZ (1∶500; MP Biomedicals (previously Cappel) Cat. No. 0855976). Secondary antibodies (donkey anti-chicken FITC, donkey anti-rabbit Cy3 and donkey anti-goat Cy3 from Jackson ImmunoResearch), were used at 1∶200 dilution. In some cases, sections were also stained with DAPI. Sections were imaged with a Nikon CCD camera or a confocal microscope (Zeiss 510).

### Genotyping

Mouse DNA was extracted and genotyping PCR performed as described previously [36].

For genotyping *M1* MADM transgenes, we used primers: KM5 (CTATGCCCGAACAACCTCTG), KM6 (ATCATATGCCAAGTACGCCC), KM7 (GGGGTCGATCTTGTCAGTCT) and KM8 (TTGCGTTGCAATTTTCTGAG). These primers amplify a 512 bp transgene fragment and a 700 bp wt *M1* locus fragment.

For genotyping *M10* MADM transgenes, we used primers: KM1 (GTGCTGCAAGGCGATTAAGT) and KM2 (TTATGTAACGCGGAACTCCA). These primers amplify a 211 bp fragment for either MADM cassette. To distinguish heterozygous vs. homozygous transgene, we used additional primers KM3 (CATATTCCAAAGCTACCACACACT) and KM4 (ATCATGGAGGAGCAGTGGAG), which amplify a 300 bp fragment from the wt *M10* locus.

*H11* MADM transgenes (available at The Jackson Laboratory: MADM-11-GT, stock# 013749 and MADM-11-TG, stock# 013751) were genotyped as described [14], using primers: SH176 (TGGAGGAGGACAAACTGGTCAC), SH177 (TCAATGGGCGGGGGTCGTT), SH178 (TTCCCTTTCTGCTTCATCTTGC) according to the genotyping protocol deposited to The Jackson Laboratory.

For genotyping *Rosa26* knock-ins we used primers Rosa4 (TCAATGGGCGGGGGTCGTT), Rosa10 (CTCTGCTGCCTCCTGGCTTCT) and Rosa11 (CGAGGCGGATCACAAGCAATA). These primers amplify a 250 bp knock-in fragment and a 330 bp wt *Rosa26* locus fragment.

For genotyping *TRE-KZ*, we used primers Tau1 (GGTGGCAAGGTGCAGATAAT) and Tau2 (CAGCTTGTGGGTTTCGATCT) to amplify a 315 bp tau fragment. We combined them with primers IMR0015 (CAAATGTTGCTTGTCTGGTG) and IMR0016 (GTCAGTCGAGTGCACAGTTT) to amplify a 200 bp internal control fragment.

For genotyping *Foxg1^tTA^*, we used primers Ling40 (TCTGCACCTTGGTGATCAAA) and Ling57 (ATCGCGATGGAGCAAAAGTA) to amplify a 270-bp fragment of tTA. We combined them with primers Globin1 (CCAATCTGCTCACACAGGATAGAGAGGGCAGG) and Globin2 (CCTTGAGGCTGTCCAAGTGATTCAGGCCATCG) to amplify a 500 bp internal control fragment.

For genotyping Cre transgenes, we used Foxg1-Cre-A (CACCCTGTTACGTATAGCCG) and Foxg1-Cre-B (GAGTCATCCTTAGCGCCGTA) to amplify a 300 bp transgene fragment and internal Globin primers as described for tTA genotyping to amplify the internal control.

Genotyping for the presence of the neomycin resistance gene (*Neo*) was performed using primers IMR3742 (GTGAGCTGCACTTCCAGAAG), IMR3743 (GACTTTCGGCATGTGAAATG), IMR013 (CTTGGGTGGAGAGGCTATTC) and IMR014 (AGGTGAGATGACAGGAGATC). These primers produce a 280 bp *Neo* band and a 180 bp wt band.

### Mouse maintenance and crosses

All mice were kept in a mixed background. All mouse lines contained some 129 and CD1 strain backgrounds, and some additionally contained C57Bl/6 and FVB. We preferred to keep mice with as much CD1 background as possible to increase fecundity.

We kept *GT* and *TG* stocks separately from each other. This approach prevents mixing up the stocks and allowed us to use the same PCR for genotyping either stock using the common pairs of primers. Other transgenes were crossed into one of the MADM cassette alleles. Once a *Nestin-Cre* or *Hprt^Cre^* line is crossed into one of the MADM cassette strains, the *loxP*-flanked (floxed) *Neo* is removed in the germline. Therefore any double positive animal of this type will transmit to its progeny the MADM cassette with removed *Neo*. The MADM cassette alleles were then usually homozygosed during maintenance to obviate the need for genotyping for that allele. For example, to generate the experimental animals *R26^TG/GT^*;*Nestin-Cre^+/−^*;*TRE-KZ^+/−^*, we would create two lines: *R26^GT/GT^* and *R26^TG/TG^*;*Nestin-Cre^+/−^*;*TRE-KZ^+/−^*. The first line, as well as other MADM-cassette lines, were usually kept homozygous (no genotyping required). In the case of *R26^GT/GT^* only, some homozygous males show decreased fertility, so from time to time a homozygous female was crossed to a CD1 wt male, and after that the homozygous stock was reestablished by crossing heterozygous mice to each other. The second line was created by sequentially introducing *Nestin-Cre* and *TRE-KZ* transgenes into *R26^TG^* mice. After the triple-transgenic mice *R26^TG/+^*;*Nestin-Cre^+/−^*;*TRE-KZ^+/−^* were created, they were crossed to *R26^TG/TG^* to create *R26^TG/TG^*;*Nestin-Cre^+/−^*;*TRE-KZ^+/−^*. These mice were maintained by crossing to *R26^TG/TG^* homozygous stock and genotyping only for the presence of *Cre* and tau.

All *GG* and *TT* alleles were generated by Cre-mediated interchromosomal recombination and were detected by screening tail samples for expression of GFP or tdT under the fluorescence microscope. In addition, under UV light, these animals appeared uniformly green and red, respectively. All *GG* and *TT* alleles had lost the floxed *Neo* from the intron as confirmed by *Neo* PCR (see Genotyping).

*Hprt^Cre^* is located on the X chromosome [19]. If maximal level of recombination is desired, it is recommended to use males for phenotypic analysis (*Hprt^Cre^/Y* as opposed *Hprt^Cre/+^*). In females, due to the random X inactivation, only roughly half of the cells have the active *Hprt^Cre^* allele.

### Reagent availability

The DNA constructs described in this paper will be deposited to Addgene. We will also deposit the following lines to The Jackson Laboratory; *R26^GT^* (stock# 017912), *R26^TG^* (stock# 017921), *R26^TT^* (stock# 017922), *R26^G-tTA2^* (stock# 017909), *Miya10^GT^* (stock# 017923), and *Miya10^TG^* (stock# 017932). Note that we have already deposited the following lines to The Jackson Laboratory: *Rosa26^GG^* (also called *MADM-GG*; stock# 006053) [10], *Hipp11^GT^* (also called *MADM-11^GT^*; stock# 013749), and *Hipp11^TG^* (also called, *MADM-11^TG^*; stock# 013751) [14]. Whereas the *GT* and *TG* mice can be used for MADM analysis of genes located on those specific chromosomes, *GG* and *TT* mice express high-level green or red fluorescence proteins globally and can be used, for example, as tissue donors in transplantation or chimeragenesis experiments.

## Supporting Information

Figure S1 **Loci and alleles used in this study.** **A**) *Miya1 (M1)* on Chr. 1; **B**) *Rosa26 (R26)* on Chr. 6; **C**) *Miya10 (M10)* on Chr. 10 and **D**) *Hipp11 (H11)* on Chr. 11. Panel D is modified after [14], where *H11^GT^* and *H11^TG^* were referred to as *MADM-11^GT^* and *MADM-11^TG^*, respectively. The *loxP*-flanked (floxed) *Neo* in any of the alleles above is converted into a single wild-type *loxP* site after the allele is crossed to a germline-expressed Cre transgene (*Nestin-Cre* or *Hprt^Cre^*). All *GG* and *TT* alleles described here were created by Cre-mediated interchromosomal recombination in meiosis and have lost the floxed *Neo*. The previously described *R26^GG^* allele (also referred to as *MADM-GG*), which was created by targeted knock-in, contains the floxed *Neo* in the intron [10].
(TIF)

Figure S2 **A scheme for generation of translocations and aneuploidy using MADM.** **A**) A cell containing Cre and two non-homologous chromosomes with reciprocal cassettes in the same orientation, e.g., Chr. 6 and Chr. 10, can generate cells containing the reciprocal translocation or aneuploidy. **B**) A cell containing Cre and two non-homologous chromosomes with reciprocal cassettes in the opposite orientation, e.g., Chr. 10 and Chr. 11, can generate cells with acentric and dicentric chromosomes and aneuploidy. In this case, change in labeling and genotype could result from the loss of acentric or dicentric chromosomes during cell division.
(TIF)

Figure S3 **A built-in** ***TRE*** **reporter within the** ***G-TET*** **allele is mostly silent.** **A**) With the aim of simplifying the use of MADM-Tet by minimizing the number of transgenes that need to be combined in a single animal, we generated another version of *G-tTA2* that had a built-in *TRE* reporter (*TRE-tdT-3Myc*), which we call *G-TET* (**Figure S1B**). In the *G-TET* construct, we flanked the *TRE* expression unit with pairs of insulators to decrease the tTA-independent leakiness of *TRE*. This leakiness was initially observed in transient transfection experiments with a plasmid containing a *pCA*-containing unit preceding the *TRE* unit (*pBT267*). This leakiness was significantly decreased when insulators were inserted to flank the *TRE* (*pBT268*, data not shown). We tested the *G-TET* construct *in vivo* by creating a knock-in mouse in *Rosa26* and then by creating a triple-transgenic mouse: *R26^TG/G-TET^*;*Nestin-Cre^+/−^*. We observed only GFP expression. The panel shows an epifluorescence image of a cortical tissue section from the genotype indicated on top, stained with anti-GFP and anti-Myc antibodies, and DAPI. **B**) To test for TRE activation in the brain, we crossed *R26^G-TET^* to *Foxg1^tTA^* knock-in allele, which expresses tTA strongly in the mouse forebrain [53] and is capable of activating a *TRE* line previously generated in our lab by random transgenesis (*TRE-SG-T*; [33]). However, when *G-TET* was crossed to *Foxg1^tTA^*, the activation was observed only in a subset of vomeronasal receptor neurons in a tTA-dependent manner. The panel shows native tdT fluorescence in forebrain tissue sections with genotypes indicated on top. Thus, we conclude that our *TRE-tdT-3Myc*, which is part of *G-TET*, cannot be activated by tTA in most cells of the forebrain.
(TIF)

## References

[1] Sternberg N, Hamilton D (1981). Bacteriophage P1 site-specific recombination. I. Recombination between loxP sites.. J Mol Biol.

[2] Broach JR, Guarascio VR, Jayaram M (1982). Recombination within the yeast plasmid 2mu circle is site-specific.. Cell.

[3] Branda CS, Dymecki SM (2004). Talking about a revolution: The impact of site-specific recombinases on genetic analyses in mice.. Dev Cell.

[4] Golic KG (1991). Site-specific recombination between homologous chromosomes in Drosophila.. Science.

[5] Xu T, Rubin GM (1993). Analysis of genetic mosaics in developing and adult Drosophila tissues.. Development.

[6] Lee T, Luo L (1999). Mosaic analysis with a repressible cell marker for studies of gene function in neuronal morphogenesis.. Neuron.

[7] Lai SL, Lee T (2006). Genetic mosaic with dual binary transcriptional systems in Drosophila.. Nat Neurosci.

[8] Griffin R, Sustar A, Bonvin M, Binari R, del Valle Rodriguez A (2009). The twin spot generator for differential Drosophila lineage analysis.. Nat Methods.

[9] Potter CJ, Tasic B, Russler EV, Liang L, Luo L (2010). The Q system: a repressible binary system for transgene expression, lineage tracing, and mosaic analysis.. Cell.

[10] Zong H, Espinosa JS, Su HH, Muzumdar MD, Luo L (2005). Mosaic analysis with double markers in mice.. Cell.

[11] Espinosa JS, Luo L (2008). Timing neurogenesis and differentiation: insights from quantitative clonal analyses of cerebellar granule cells.. J Neurosci.

[12] Muzumdar MD, Luo L, Zong H (2007). Modeling sporadic loss of heterozygosity in mice by using mosaic analysis with double markers (MADM).. Proc Natl Acad Sci U S A.

[13] Espinosa JS, Wheeler DG, Tsien RW, Luo L (2009). Uncoupling dendrite growth and patterning: single-cell knockout analysis of NMDA receptor 2B.. Neuron.

[14] Hippenmeyer S, Youn YH, Moon HM, Miyamichi K, Zong H (2010). Genetic mosaic dissection of Lis1 and Ndel1 in neuronal migration.. Neuron.

[15] Liu C, Sage JC, Miller MR, Verhaak RG, Hippenmeyer S (2011). Mosaic Analysis with Double Markers Reveals Tumor Cell of Origin in Glioma.. Cell.

[16] Shaner NC, Campbell RE, Steinbach PA, Giepmans BN, Palmer AE (2004). Improved monomeric red, orange and yellow fluorescent proteins derived from Discosoma sp. red fluorescent protein.. Nat Biotechnol.

[17] Urlinger S, Baron U, Thellmann M, Hasan MT, Bujard H (2000). Exploring the sequence space for tetracycline-dependent transcriptional activators: novel mutations yield expanded range and sensitivity.. Proc Natl Acad Sci U S A.

[18] Petersen PH, Zou K, Hwang JK, Jan YN, Zhong W (2002). Progenitor cell maintenance requires numb and numblike during mouse neurogenesis.. Nature.

[19] Tang SH, Silva FJ, Tsark WM, Mann JR (2002). A Cre/loxP-deleter transgenic line in mouse strain 129S1/SvImJ.. Genesis.

[20] Seibler J, Schubeler D, Fiering S, Groudine M, Bode J (1998). DNA cassette exchange in ES cells mediated by Flp recombinase: an efficient strategy for repeated modification of tagged loci by marker-free constructs.. Biochemistry.

[21] Horn C, Hansen J, Schnutgen F, Seisenberger C, Floss T (2007). Splinkerette PCR for more efficient characterization of gene trap events.. Nat Genet.

[22] Doetschman T, Gregg RG, Maeda N, Hooper ML, Melton DW (1987). Targetted correction of a mutant HPRT gene in mouse embryonic stem cells.. Nature.

[23] Thomas KR, Capecchi MR (1987). Site-directed mutagenesis by gene targeting in mouse embryo-derived stem cells.. Cell.

[24] Joyner AL (2000). Gene Targeting, A Practical Approach.

[25] Muzumdar MD, Tasic B, Miyamichi K, Li L, Luo L (2007). A global double-fluorescent Cre reporter mouse.. Genesis.

[26] Soriano P (1999). Generalized lacZ expression with the ROSA26 Cre reporter strain.. Nat Genet.

[27] Van Deursen J, Fornerod M, Van Rees B, Grosveld G (1995). Cre-mediated site-specific translocation between nonhomologous mouse chromosomes.. Proc Natl Acad Sci U S A.

[28] Smith AJ, De Sousa MA, Kwabi-Addo B, Heppell-Parton A, Impey H (1995). A site-directed chromosomal translocation induced in embryonic stem cells by Cre-loxP recombination.. Nat Genet.

[29] Collins EC, Pannell R, Simpson EM, Forster A, Rabbitts TH (2000). Inter-chromosomal recombination of Mll and Af9 genes mediated by cre-loxP in mouse development.. EMBO Rep.

[30] Buchholz F, Refaeli Y, Trumpp A, Bishop JM (2000). Inducible chromosomal translocation of AML1 and ETO genes through Cre/loxP-mediated recombination in the mouse.. EMBO Rep.

[31] Forster A, Pannell R, Drynan LF, McCormack M, Collins EC (2003). Engineering de novo reciprocal chromosomal translocations associated with Mll to replicate primary events of human cancer.. Cancer Cell.

[32] Drynan LF, Pannell R, Forster A, Chan NM, Cano F (2005). Mll fusions generated by Cre-loxP-mediated de novo translocations can induce lineage reassignment in tumorigenesis.. EMBO J.

[33] Li L, Tasic B, Micheva KD, Ivanov VM, Spletter ML (2010). Visualizing the distribution of synapses from individual neurons in the mouse brain.. PLoS One.

[34] Luo L (2007). Fly MARCM and mouse MADM: genetic methods of labeling and manipulating single neurons.. Brain Res Rev.

[35] Yu CR, Power J, Barnea G, O'Donnell S, Brown HE (2004). Spontaneous neural activity is required for the establishment and maintenance of the olfactory sensory map.. Neuron.

[36] Tasic B, Hippenmeyer S, Wang C, Gamboa M, Zong H (2011). Site-specific integrase-mediated transgenesis in mice via pronuclear injection.. Proc Natl Acad Sci U S A.

[37] Townes TM, Lingrel JB, Chen HY, Brinster RL, Palmiter RD (1985). Erythroid-specific expression of human beta-globin genes in transgenic mice.. EMBO J.

[38] Chen ZY, He CY, Ehrhardt A, Kay MA (2003). Minicircle DNA vectors devoid of bacterial DNA result in persistent and high-level transgene expression in vivo.. Mol Ther.

[39] Liu P, Jenkins NA, Copeland NG (2002). Efficient Cre-loxP-induced mitotic recombination in mouse embryonic stem cells.. Nat Genet.

[40] Pankiewicz R, Karlen Y, Imhof MO, Mermod N (2005). Reversal of the silencing of tetracycline-controlled genes requires the coordinate action of distinctly acting transcription factors.. J Gene Med.

[41] Zhu P, Aller MI, Baron U, Cambridge S, Bausen M (2007). Silencing and un-silencing of tetracycline-controlled genes in neurons.. PLoS One.

[42] Zeng H, Horie K, Madisen L, Pavlova MN, Gragerova G (2008). An inducible and reversible mouse genetic rescue system.. PLoS Genet.

[43] Novak A, Guo C, Yang W, Nagy A, Lobe CG (2000). Z/EG, a double reporter mouse line that expresses enhanced green fluorescent protein upon Cre-mediated excision.. Genesis.

[44] Seibler J, Bode J (1997). Double-reciprocal crossover mediated by FLP-recombinase: a concept and an assay.. Biochemistry.

[45] Groth AC, Olivares EC, Thyagarajan B, Calos MP (2000). A phage integrase directs efficient site-specific integration in human cells.. Proc Natl Acad Sci U S A.

[46] Lee G, Saito I (1998). Role of nucleotide sequences of loxP spacer region in Cre-mediated recombination.. Gene.

[47] Miyamichi K, Amat F, Moussavi F, Wang C, Wickersham I (2011). Cortical representations of olfactory input by trans-synaptic tracing.. Nature.

[48] Srinivas S, Watanabe T, Lin CS, William CM, Tanabe Y (2001). Cre reporter strains produced by targeted insertion of EYFP and ECFP into the ROSA26 locus.. BMC Dev Biol.

[49] Okada A, Lansford R, Weimann JM, Fraser SE, McConnell SK (1999). Imaging cells in the developing nervous system with retrovirus expressing modified green fluorescent protein.. Exp Neurol.

[50] Siemering KR, Golbik R, Sever R, Haseloff J (1996). Mutations that suppress the thermosensitivity of green fluorescent protein.. Curr Biol.

[51] Nagy A, Rossant J, Nagy R, Abramow-Newerly W, Roder JC (1993). Derivation of completely cell culture-derived mice from early-passage embryonic stem cells.. Proc Natl Acad Sci U S A.

[52] Raymond CS, Soriano P (2007). High-efficiency FLP and PhiC31 site-specific recombination in mammalian cells.. PLoS One.

[53] Hanashima C, Li SC, Shen L, Lai E, Fishell G (2004). Foxg1 suppresses early cortical cell fate.. Science.

