# Correction: Extensions of MADM (Mosaic Analysis with Double Markers) in Mice

**DOI:** 10.1371/annotation/e4275a34-48e1-42b8-8615-f59aacaf3733

**Published:** 2012-07-10

**Authors:** Bosiljka Tasic, Kazunari Miyamichi, Simon Hippenmeyer, Vardhan S. Dani, Hong Zeng, William Joo, Hui Zong, Yanru Chen-Tsai, Liqun Luo

There was an error in the corresponding author designation. Bosiljka Tasic and Liqun Luo are both corresponding authors. Bosiljka Tasic's e-mail address is bosiljka.tasic@gmail.com. Liqun Luo's published e-mail address was incorrect; lluo@stanford.edu is correct. 

