# Supplementary figures and images for: Extensions of MADM (Mosaic Analysis with Double Markers) in Mice
Source: PLoS One. 2012 Mar 27;7(3):e33332. doi: 10.1371/journal.pone.0033332 (PMC3314016; doi:10.1371/journal.pone.0033332)

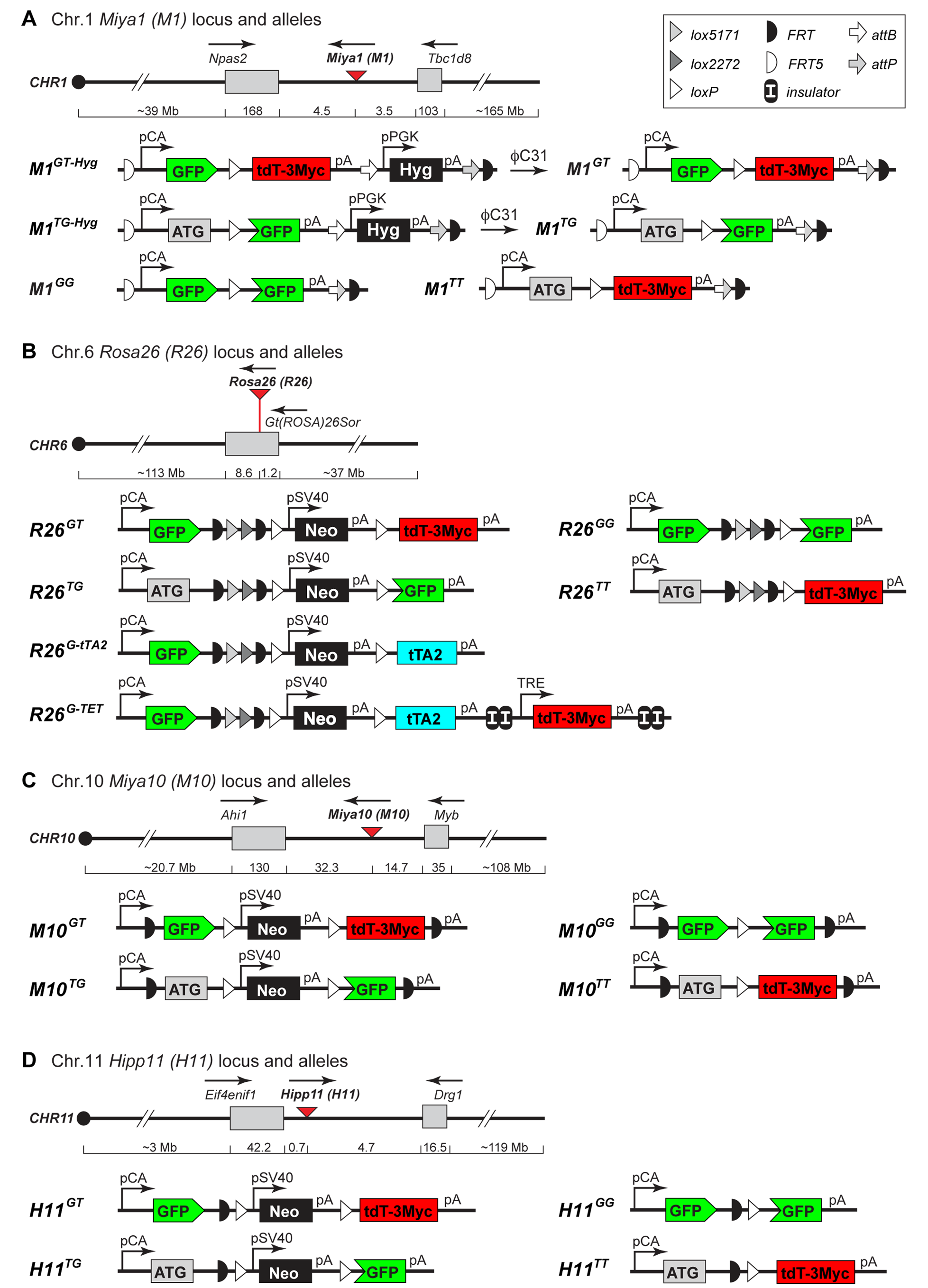

Supplement: Figure S1 — Loci and alleles used in this study. A) Miya1 (M1) on Chr. 1; B) Rosa26 (R26) on Chr. 6; C) Miya10 (M10) on Chr. 10 and D) Hipp11 (H11) on Chr. 11. Panel D is modified after [14], where H11GT and H11TG were referred to as MADM-11GT and MADM-11TG, respectively. The loxP-flanked (floxed) Neo in any of the alleles above is converted into a single wild-type loxP site after the allele is crossed to a germline-expressed Cre transgene (Nestin-Cre or HprtCre). All GG and TT alleles described here were created by Cre-mediated interchromosomal recombination in meiosis and have lost the floxed Neo. The previously described R26GG allele (also referred to as MADM-GG), which was created by targeted knock-in, contains the floxed Neo in the intron [10]. (TIF) [file pone.0033332.s001.tif]

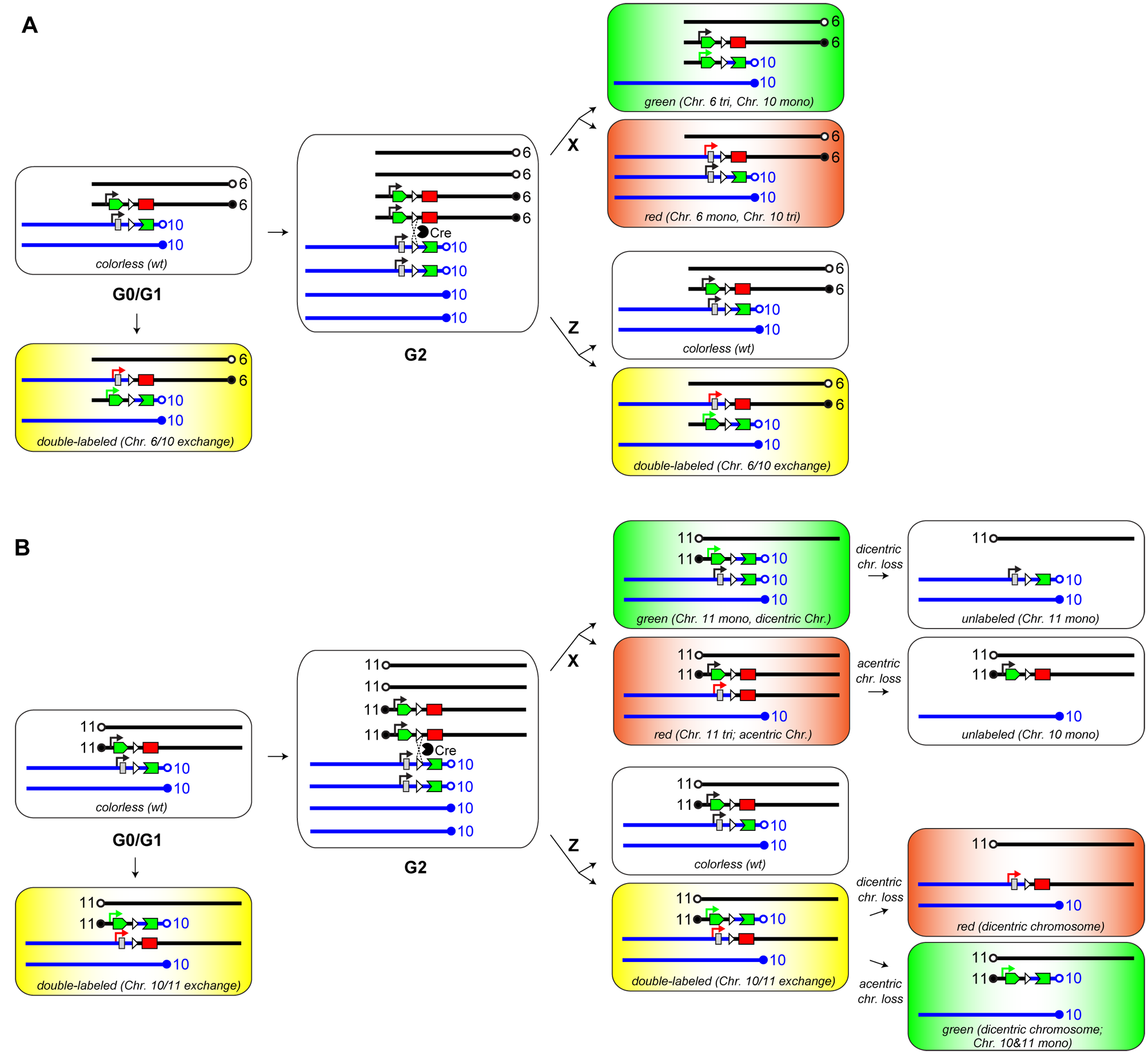

Supplement: Figure S2 — A scheme for generation of translocations and aneuploidy using MADM. A) A cell containing Cre and two non-homologous chromosomes with reciprocal cassettes in the same orientation, e.g., Chr. 6 and Chr. 10, can generate cells containing the reciprocal translocation or aneuploidy. B) A cell containing Cre and two non-homologous chromosomes with reciprocal cassettes in the opposite orientation, e.g., Chr. 10 and Chr. 11, can generate cells with acentric and dicentric chromosomes and aneuploidy. In this case, change in labeling and genotype could result from the loss of acentric or dicentric chromosomes during cell division. (TIF) [file pone.0033332.s002.tif]

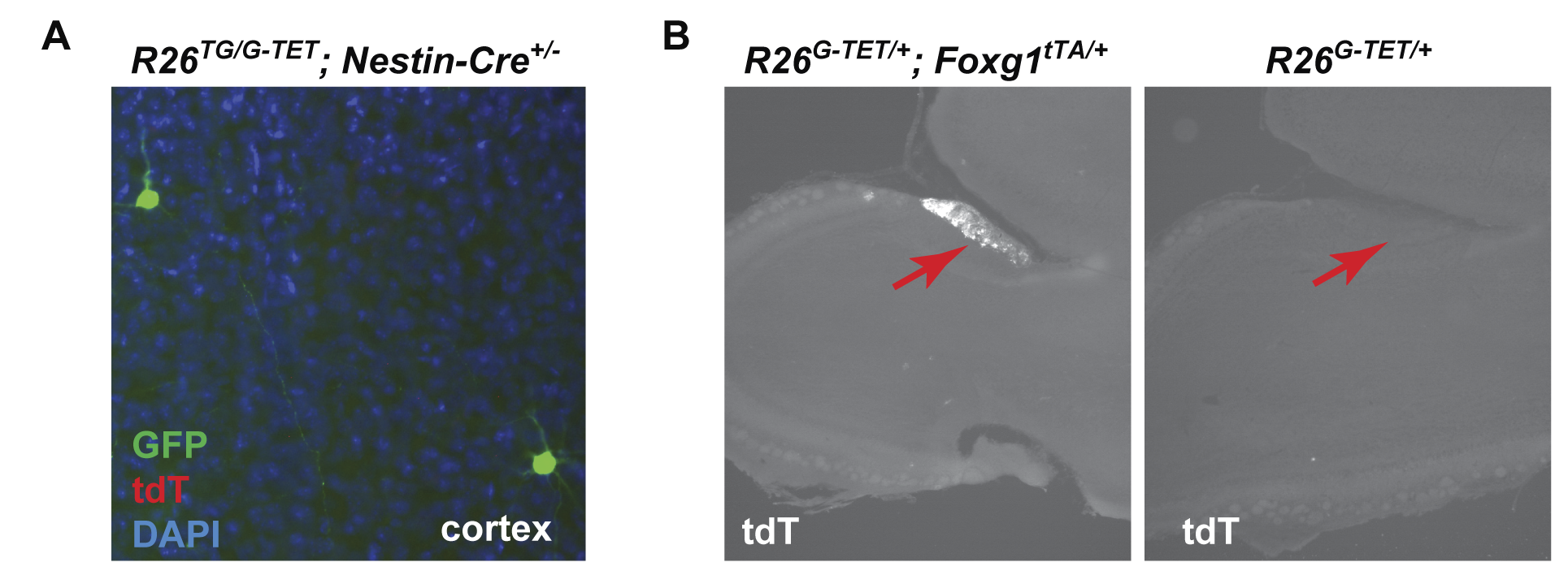

Supplement: Figure S3 — A built-in TRE reporter within the G-TET allele is mostly silent. A) With the aim of simplifying the use of MADM-Tet by minimizing the number of transgenes that need to be combined in a single animal, we generated another version of G-tTA2 that had a built-in TRE reporter (TRE-tdT-3Myc), which we call G-TET (Figure S1B). In the G-TET construct, we flanked the TRE expression unit with pairs of insulators to decrease the tTA-independent leakiness of TRE. This leakiness was initially observed in transient transfection experiments with a plasmid containing a pCA-containing unit preceding the TRE unit (pBT267). This leakiness was significantly decreased when insulators were inserted to flank the TRE (pBT268, data not shown). We tested the G-TET construct in vivo by creating a knock-in mouse in Rosa26 and then by creating a triple-transgenic mouse: R26TG/G-TET;Nestin-Cre+/−. We observed only GFP expression. The panel shows an epifluorescence image of a cortical tissue section from the genotype indicated on top, stained with anti-GFP and anti-Myc antibodies, and DAPI. B) To test for TRE activation in the brain, we crossed R26G-TET to Foxg1tTA knock-in allele, which expresses tTA strongly in the mouse forebrain [53] and is capable of activating a TRE line previously generated in our lab by random transgenesis (TRE-SG-T; [33]). However, when G-TET was crossed to Foxg1tTA, the activation was observed only in a subset of vomeronasal receptor neurons in a tTA-dependent manner. The panel shows native tdT fluorescence in forebrain tissue sections with genotypes indicated on top. Thus, we conclude that our TRE-tdT-3Myc, which is part of G-TET, cannot be activated by tTA in most cells of the forebrain. (TIF) [file pone.0033332.s003.tif]
